# Self-Harm, Suicidal Behaviours, and Cyberbullying in Children and Young People: Systematic Review

**DOI:** 10.2196/jmir.9044

**Published:** 2018-04-19

**Authors:** Ann John, Alexander Charles Glendenning, Amanda Marchant, Paul Montgomery, Anne Stewart, Sophie Wood, Keith Lloyd, Keith Hawton

**Affiliations:** ^1^ Population Psychiatry, Suicide and Informatics Medical School Swansea University Swansea United Kingdom; ^2^ Department of Social Policy, Sociology and Criminology School of Social Policy University of Birmingham Birmingham United Kingdom; ^3^ Oxford Central Child and Adolescent Mental Health Services Oxford Health NHS Foundation Trust Oxford United Kingdom; ^4^ Centre for Suicide Research University of Oxford Oxford United Kingdom

**Keywords:** cyberbullying, bullying, self-injurious behavior, suicide, suicide, attempted, suicidal ideation

## Abstract

**Background:**

Given the concerns about bullying via electronic communication in children and young people and its possible contribution to self-harm, we have reviewed the evidence for associations between cyberbullying involvement and self-harm or suicidal behaviors (such as suicidal ideation, suicide plans, and suicide attempts) in children and young people.

**Objective:**

The aim of this study was to systematically review the current evidence examining the association between cyberbullying involvement as victim or perpetrator and self-harm and suicidal behaviors in children and young people (younger than 25 years), and where possible, to meta-analyze data on the associations.

**Methods:**

An electronic literature search was conducted for all studies published between January 1, 1996, and February 3, 2017, across sources, including MEDLINE, Cochrane, and PsycINFO. Articles were included if the study examined any association between cyberbullying involvement and self-harm or suicidal behaviors and reported empirical data in a sample aged under 25 years. Quality of included papers was assessed and data were extracted. Meta-analyses of data were conducted.

**Results:**

A total of 33 eligible articles from 26 independent studies were included, covering a population of 156,384 children and young people. A total of 25 articles (20 independent studies, n=115,056) identified associations (negative influences) between cybervictimization and self-harm or suicidal behaviors or between perpetrating cyberbullying and suicidal behaviors. Three additional studies, in which the cyberbullying, self-harm, or suicidal behaviors measures had been combined with other measures (such as traditional bullying and mental health problems), also showed negative influences (n=44,526). A total of 5 studies showed no significant associations (n=5646). Meta-analyses, producing odds ratios (ORs) as a summary measure of effect size (eg, ratio of the odds of cyber victims who have experienced SH vs nonvictims who have experienced SH), showed that, compared with nonvictims, those who have experienced cybervictimization were OR 2.35 (95% CI 1.65-3.34) times as likely to self-harm, OR 2.10 (95% CI 1.73-2.55) times as likely to exhibit suicidal behaviors, OR 2.57 (95% CI 1.69-3.90) times more likely to attempt suicide, and OR 2.15 (95% CI 1.70-2.71) times more likely to have suicidal thoughts. Cyberbullying perpetrators were OR 1.21 (95% CI 1.02-1.44) times more likely to exhibit suicidal behaviors and OR 1.23 (95% CI 1.10-1.37) times more likely to experience suicidal ideation than nonperpetrators.

**Conclusions:**

Victims of cyberbullying are at a greater risk than nonvictims of both self-harm and suicidal behaviors. To a lesser extent, perpetrators of cyberbullying are at risk of suicidal behaviors and suicidal ideation when compared with nonperpetrators. Policy makers and schools should prioritize the inclusion of cyberbullying involvement in programs to prevent traditional bullying. Type of cyberbullying involvement, frequency, and gender should be assessed in future studies.

## Introduction

### Cyberbullying

*Bullying* is an aggressive, intentional act carried out by a group or an individual repeatedly and over time against a victim who cannot easily defend himself or herself. Traditionally, bullying could be direct—physical, verbal, or relational (eg, social exclusion)— or indirect (eg, rumor spreading) [1]. However, with the advent of electronic communication (eg, social media and instant messaging) via the internet and mobile phones, *cyberbullying* has emerged. This can be similarly defined, with the addition that it occurs via electronic forms of contact [2]. As the harassment of victims takes place electronically, the manner and timings in which they are targeted, as well as how they cope in response, and the proximity of relationships between victims and perpetrators, are uniquely different compared with traditional bullying. Cyberbullying victimization tends to occur at a later age, around 14 years, when children spend more time on their mobile phones [3] and social networking sites [4]. Perpetrators of cyberbullying have a degree of anonymity not possible in traditional bullying, and the potential exposure and embarrassment of the victim is on a larger scale. It is possible to victimize a peer within their own home or elsewhere at any time of day or night, and should they remove themselves from the site, the messages often accumulate. This presents new challenges for individuals, families, schools, professionals, researchers, and policy makers.

The adverse impact of bullying on children and young people’s lives, be they victim, perpetrator, or both, has long been recognized [1]. Being bullied is often associated with mental health problems (including depressive symptomatology), self-harm (SH), and suicidal behaviors [5-9]. A meta-analysis [7] found that traditional bullying was associated with general anxiety, with an odds ratio (OR) of 2.55 (95% CI 1.28-3.83), and depression, with OR 6.22 (95% CI 3.11-9.33). School bullying (less than weekly) has been shown to be associated with suicidal ideation (OR 2.79, 95% CI 1.64-4.75) and suicide attempt (OR 2.66, 95% CI 1.58-4.47) [9]. Some studies have found over 85% of those involved in cyberbullying are also involved in traditional bullying and have suggested that health issues associated with cyberbullying involvement are mediated through traditional bullying [10]. The reported prevalence of cyberbullying involvement varies widely across countries. This reflects societal factors, stigma, and also differing interpretations of “repeatedly and over time.” Estimates indicate that between 15% and 35% of young people have been victims of cyberbullying and between 10% and 20% of individuals admit to having cyberbullied others [11].

### Previous Literature on Cyberbullying and Self-Harm and Suicidal Behaviors

Four previous systematic reviews [12-15] have demonstrated an association between cyberbullying involvement and SH or suicidal behaviors. They included a maximum of 5 studies each; 8 in total between them with only 6 studies eligible for meta-analysis [11,16-22]. The study by Daine et al [12], which included 2 papers on this topic, concluded that cyberbullying involvement was one of the most significant negative aspects of the influence of the internet on SH but that this was an area of research still in its infancy. Given the rapid expansion of evidence in the field, the apparent rise in prevalence of SH [23], and the changing nature of electronic communication in young people, it is timely to reassess the literature.

The aim of this study was to systematically review the current evidence examining the association between cyberbullying involvement (as victim, perpetrator, or both) and SH and suicidal behaviors in children and young people (younger than 25 years). Where possible, we aimed to meta-analyze data on the associations.

## Methods

### Search Strategy

A protocol for this review was registered with PROSPERO (ID: CRD42017056487). This review was conducted in compliance with the guidelines for Meta-Analyses and Systematic Reviews of Observational Studies [24] and the Preferred Reporting Items for Systematic Reviews and Meta-Analyses guidelines [25].

A literature search was conducted for all studies published in English between January 1, 1996, and February 3, 2017. The databases searched included the Cochrane Library, Medical Literature Analysis and Retrieval System Online, PROSPERO, PsycINFO, PubMed, and Scopus. Additional searches were conducted in health improvement sources (eg, Health Evidence Canada), topic-specific websites (eg, American Association of Suicidology), and meta-search engines (Google). Gray literature was further explored by contacting experts in the field for any unreported or ongoing studies.

The following terms were searched in free text or keywords:

“Automutilation,” “Distress*,” “Emotion*,” “nssi,” “((oneself or myself or self) adj2 (cut* or harm* or hurt* or kill or injur* or mutilat*)),” “(psychological adj (stress or distress)),” “SIB', 'Suicid*,” “Aol,” “Askfm,” “Bebo,” “blog*,” “chat room* OR chatroom*,” “cyber*,” “discussion forum,” “e-communi*,” “e-material*,” “Facebook,” “google*,” “hashtag,” “image sharing,” “Instagram,” “instant messag*,” “internet*,” “live chat,” “live journal*,” “meme,” “MSN,” “Myspace,” “on line OR online,” “photo sharing,” “Pinterest,” “podcast*,” “social network*,” “spam*,” “troll*,” “Tumblr,” “tweet*,” “Twitter,” “video sharing,” “vine,” “virtual*,” “vlog*,” “web*,” and “YouTube.”

The following terms were searched alongside the following database subject headings:

*Medical subject headings*: “self-injurious behavior,” “stress, psychological,” “blogging,” “electronic mail,” “internet,” “social media,” “social networking,” “bullying,” “adolescent,” “child,” “students,” and “young adult.”

*Health Management Information Consortium*: “attempted suicide,” “self harm,” “suicide pacts,” “suicide,” “bullying,” “cyberspace,” “internet,” “internet websites,” “intranet,” and “world wide web.”

*PsycInfo*: “attempted suicide,” “self destructive behaviour,” “self injurious behavior,” “suicidal ideation,” “suicide prevention,” “cyberbullying,” and “adolescent attitudes.”

*Excerpta Medica dataBASE*: “automutilation,” “suicidal behaviour,” “suicide,” “bullying,” “internet,” “social network,” “adolescent,” “child,” and “young.”

### Study Selection

#### Inclusion and Exclusion Criteria

Included studies were those which examined any association between cyberbullying involvement (victimization or perpetration) and SH or suicidal behaviors and included empirical data in a sample aged younger than 25 years. The criteria used to determine eligibility for inclusion in the study were based on the study by Daine et al [12]. These are shown in Multimedia Appendix 1.

#### Screening

A stepwise screening process was performed independently by two researchers (AJ and AG). Initially, title and duplication manual screening was conducted. Titles with no relevance to the study were excluded. Any disagreements between reviewers’ categorizations were put forward for abstract review. In the second stage, the remaining titles and abstracts were screened. Reference lists of review articles and included articles were manually screened for relevant studies. Studies were forwarded to the third stage of full-text article screening if they met the inclusion criteria or a decision could not be made on title and abstract alone. Any disagreements were resolved by consensus with a third reviewer (AM).

Included papers that investigated cyberbullying involvement (victimization or perpetration) and SH or suicidal behaviors and reported empirical data in a sample under the age of 25 years were forwarded for detailed analysis of their methodology and content.

#### Study Quality and Data Extraction

The quality of included papers was assessed independently by two reviewers (AG and AM) using the Critical Appraisal Skills Programme [26]. This program assesses multiple aspects of each paper in detail, including study design, representativeness of sample, bias, aspects of data collection, use of validated outcome measures, and whether conclusions reflect results. A quality rating of low, medium, or high was obtained using these quality standards as per Daine et al [12].

A data extraction sheet, developed by Daine et al [12], was adapted and used to record specific findings; identify themes; and ascertain potential biases, limitations, and weaknesses. Data were extracted independently by two reviewers (AG and AM). In particular, data on the type of bullying involvement (victim or perpetrator) and types of outcome measures were recorded. The latter were measures of SH, suicide, and other suicidal behaviors including suicide attempts and suicidal ideation, as recorded in the study. SH was defined as an intentional act of nonfatal self-injury or self-poisoning, regardless of intent or motivation [27]. Suicidal behaviors included thoughts of suicide or suicidal ideation, suicide plans, and suicide attempts [28]. Other outcomes such as mental health problems and traditional bullying were only extracted if it was possible to distinguish their relationship to cyberbullying involvement and SH or suicidal behaviors. Any disagreements at this stage were resolved by consensus with a third reviewer (AJ). Data were stored in Statistical Package for the Social Sciences (SPSS) version 22 (IBM Corp).

### Prevalence

All available data were collected on the prevalence rates of cybervictimization for the total study population. Weighted prevalence rates were then calculated based on the differing populations of each study [29]. Individual study weights were calculated using each study population total with respect to the sum total of all study populations eligible for inclusion. Subsequently, it was possible to calculate a simple weighted average for the overall prevalence. Studies that did not include data on prevalence or where the prevalence was based on one sex only were excluded from the overall weighted calculation.

### Meta-Analysis

Studies with outcomes relating to SH, suicidal behaviors, suicide attempts, and suicidal ideation were assessed for suitability for meta-analysis. Decisions on the appropriateness of meta-analysis were based on consistency of outcome measures and level of heterogeneity between studies.

The common effect size index, the log of the OR, was used in the meta-analysis. Other types of effect sizes were transformed into this before the analysis. Inclusion criteria for the effect size index were based on the recommendations of Borenstein et al [30]. Reanalyses of raw data or conversions were performed only when necessary. Studies that did not include measures of precision with their results, that is, a corresponding CI or *P* value, were excluded from meta-analysis as these are required to calculate corresponding variances [31]. Final results were transformed from the log of the OR to the OR for presentation. The OR is here defined as the ratio between the odds of an individual who is involved in cyberbullying having experienced SH or suicidal behaviors and the odds of an individual who is not involved in cyberbullying having experienced SH or suicidal behaviors.

Where a study presented more than one effect size eligible for a meta-analysis, the most appropriate measure to maintain homogeneity of outcomes was included, for example, “suicide attempt” was chosen over “suicide attempt requiring medical treatment.” However, where it was not possible to make such a distinction between two eligible outcomes (eg, female and male populations), the effect sizes were combined as an average based on the recommendations of Borenstein et al [30]. If a study presented results in such a way that it was not possible to disaggregate outcomes of interest from other measures not considered in this review (eg, combined with mental health problems or with other forms of bullying), then the study was excluded from meta-analysis. Where two or more studies based on the same study population were eligible for meta-analysis, the study with the greatest sample size was included. Where a study presented results in terms of a range of frequencies (eg, “rarely,” “sometimes,” and “often”), the best average fit was chosen, that is, “sometimes” would be chosen over “rarely” or “often” for inclusion. Further details of the method are available in Multimedia Appendix 2.

Meta-analysis was performed using Matlab R2015a. The DerSimonian and Laird random-effects model was employed. Forest plots, summary effect sizes, CIs, *P* values, and measures of heterogeneity in the form of the *Q*- and I^2^-statistics were calculated. The I^2^-statistic was interpreted as per Higgins et al [32]: low (25%≤I^2^<50%), moderate (50%≤I^2^<75%), and high (I^2^_ _≥75%).

Meta-regressions, sensitivity analyses, and funnel plots were conducted to assess the effects of potential confounders, where relevant, and publication bias, where the number of eligible studies allowed for a robust assessment. The methods used are described in Multimedia Appendix 2.

## Results

**Figure 1:**
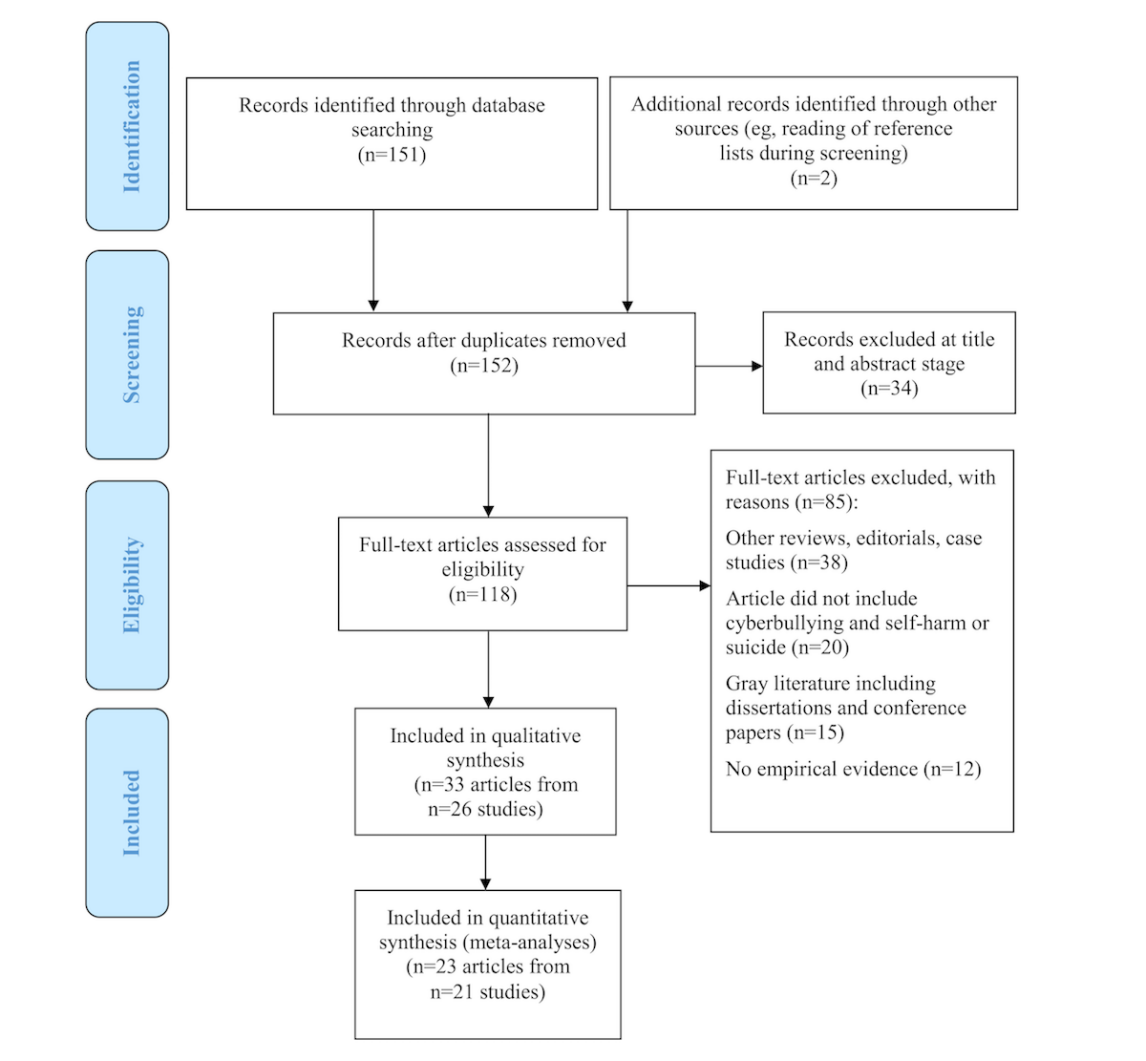
Preferred Reporting Items for Systematic Reviews and Meta-Analyses (PRISMA) flowchart displaying the different stages of the screening process.

In total, 153 citations were identified by all electronic searches. A flowchart of the results of the search strategy and screening process is detailed in Figure 1.

### Description of Included Studies

A total of 33 articles were eligible for inclusion in the review and forwarded for data extraction, comprising 26 independent studies and 156,384 individual participants. In total, 19 studies were from the United States; 7 from Canada; 1 each from Belgium, the Netherlands, Taiwan, Hong Kong, South Korea, and Australia; and 1 study that was conducted based on data from a combination of 24 different European nations [33]. All papers were based on observational studies: 28 cross-sectional based on survey data, 3 case-control studies, 1 cohort study [34], and 1 ecological study [33]. Multimedia Appendix 3 summarizes the aims, quality ratings, and findings of the included articles.

#### Study Populations

Of the 26 independent studies (33 articles), 20 were based on unique populations, whereas 6 independent studies (13 articles) had populations shared by at least one other article. Those articles that shared study populations were as follows: Schenk et al [22,35]; Bauman et al [18] and Romero et al [36]; Alavi et al [37] and Roberts et al [38]; Cénat et al [39] and Hébert et al [40]; Hay and Meldrum [16] and Hay et al [41]; and Messias et al [42], Reed et al [43], and Kindrick et al [44]. Further details of these study populations are available in Multimedia Appendix 4.

Excluding duplicate populations [35-37,39,41,43,44], the total number of unique participants was 156,384, with a mean of 6015 and median of 2243 individuals per study. Most studies included both female and male participants (often not reported separately). However, 2 studies included one sex only [36,45]. The youngest reported mean participant age was 12.5 years [34], whereas the oldest was 20.0 years [22,35].

#### Cyberbullying Involvement

Cybervictimization was analyzed in 25 included studies [16,17,20,22,33,34,37-44,46-56]; 7 studies examined both cybervictimization and cyberbullying perpetration [11, 18,19,21,36,45,57], and 1 study investigated cyberbullying perpetration, but also included those who were both victims and perpetrators [35]. Inclusion in one of these groups was most commonly assessed by a participant’s yes or no response to a single question. For example, 7 studies used a question from the Youth Risk Behavior Survey: “During the past 12 months, have you ever been electronically bullied? (include being bullied through email, chat rooms, instant messaging, websites, or texting).” A total of 29 studies were based on self-report questionnaires, 2 on researcher-completed ones [33,52], and 2 on retrospective reviews of patients’ medical records [37,38].

Only 4 studies [11,19-21] reported the medium through which cyberbullying (victimization and perpetration) occurred. The three most commonly experienced forms of victimization reported by Hinduja and Patchin [11] were as follows: “email” (18.3%), “instant message” (16.0%), and “MySpace” (14.2%), whereas the most common forms of perpetration were as follows: “posted something online about another person to make others laugh” (23.1%), “sent someone a computer text message to make them angry or to make fun of them” (13.7%), and “took a picture of someone and posted it online without their permission” (12.1%). Goebert et al [20] reported wide ranges of the medium of cybervictimization used across different Asian and Pacific Islander ethnic groups, for example, “text” (18.5%-27.8%). No direct associations could be calculated between medium of cyberbullying involvement and the SH or suicidal outcomes because of the way the data were collected and presented. Multimedia Appendix 5 displays the measures used in all 33 studies.

The findings of Elgar et al [49] and Kodish et al [50] suggested that the health consequences of cybervictimization are not completely attributable to its co-occurrence with face-to-face bullying. Similarly, the correlations reported in Fu et al [33] between cybervictimization and unnatural child deaths were independent of traditional bullying.

#### Prevalence of Cybervictimization

On the basis of 20 eligible studies (116,433 individuals), 12.6% (95% CI 12.4%-12.7%) of individuals had experienced cybervictimization. Weighted prevalence and prevalence by study are shown in Multimedia Appendix 6.

#### Outcomes

Measures of SH were assessed in 19 articles (14 independent studies), suicidal behaviors in 32 articles (25 independent studies), suicide attempts in 16 articles (12 independent studies), and suicidal ideation in 27 articles (20 independent studies). One study [52] measured “thoughts of self-harm.” No studies included death by suicide as an outcome, but 1 study [33] explored the association between cybervictimization and unnatural child death, which included suicides, accidental deaths, and death by assault. Outcomes and measures used are included in Multimedia Appendix 5.

### Associations Between Cyberbullying Involvement and Self-Harm and Suicidal Behaviors

A total of 25 articles reported a positive association or negative influence of cyberbullying involvement (victimization or perpetration) on SH and suicidal behaviors; 3 [33,40,53] found negative influences in analyses in which the cyberbullying, SH, or suicidal behaviors measures had been combined with other measures; and 5 [19,21,36,52,56] found no significant association (2 of these articles [19,21] reported the proportion of cyber victims who had experienced SH and suicidal behaviors, but not that of nonvictims, meaning that no association could be determined).

**Figure 2:**
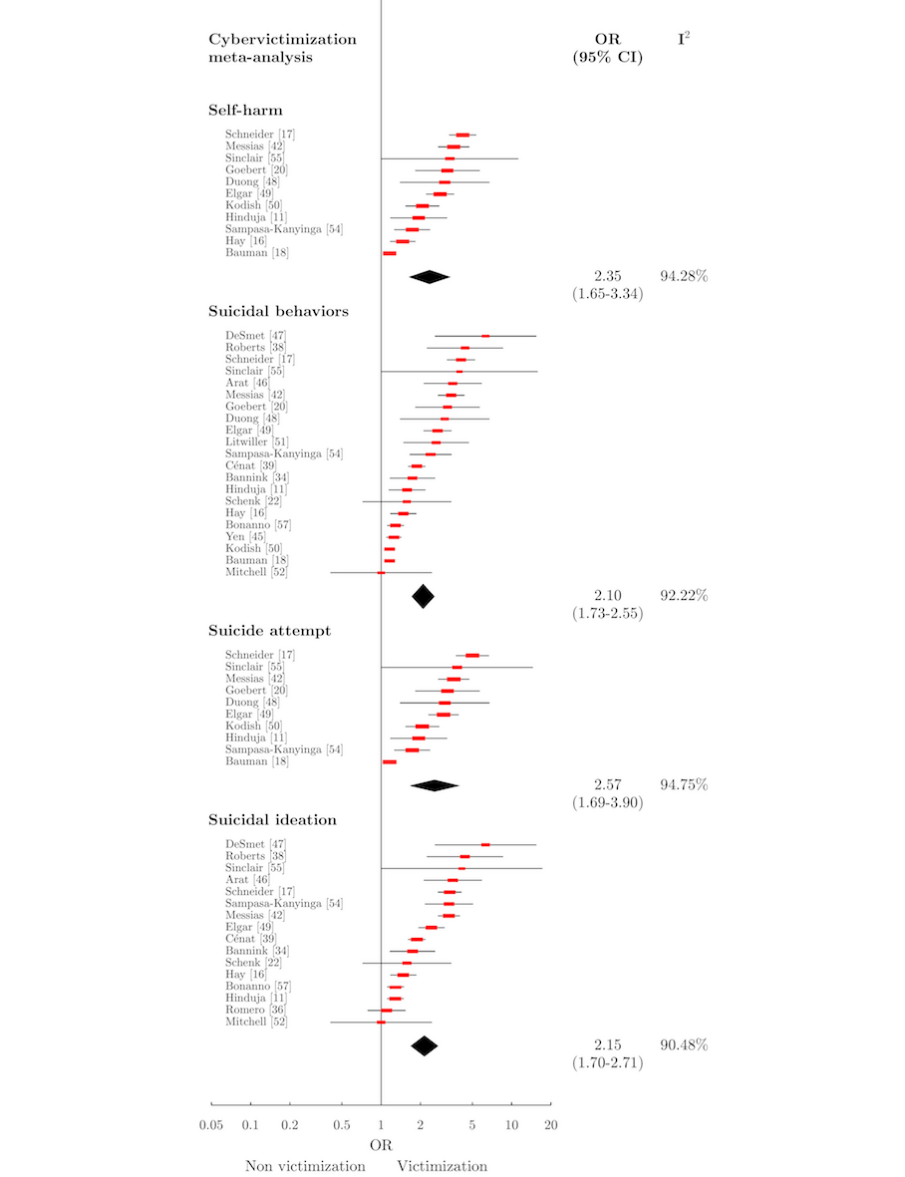
Forest plot of cybervictimization meta-analyses. OR: odds ratio.

**Figure 3:**
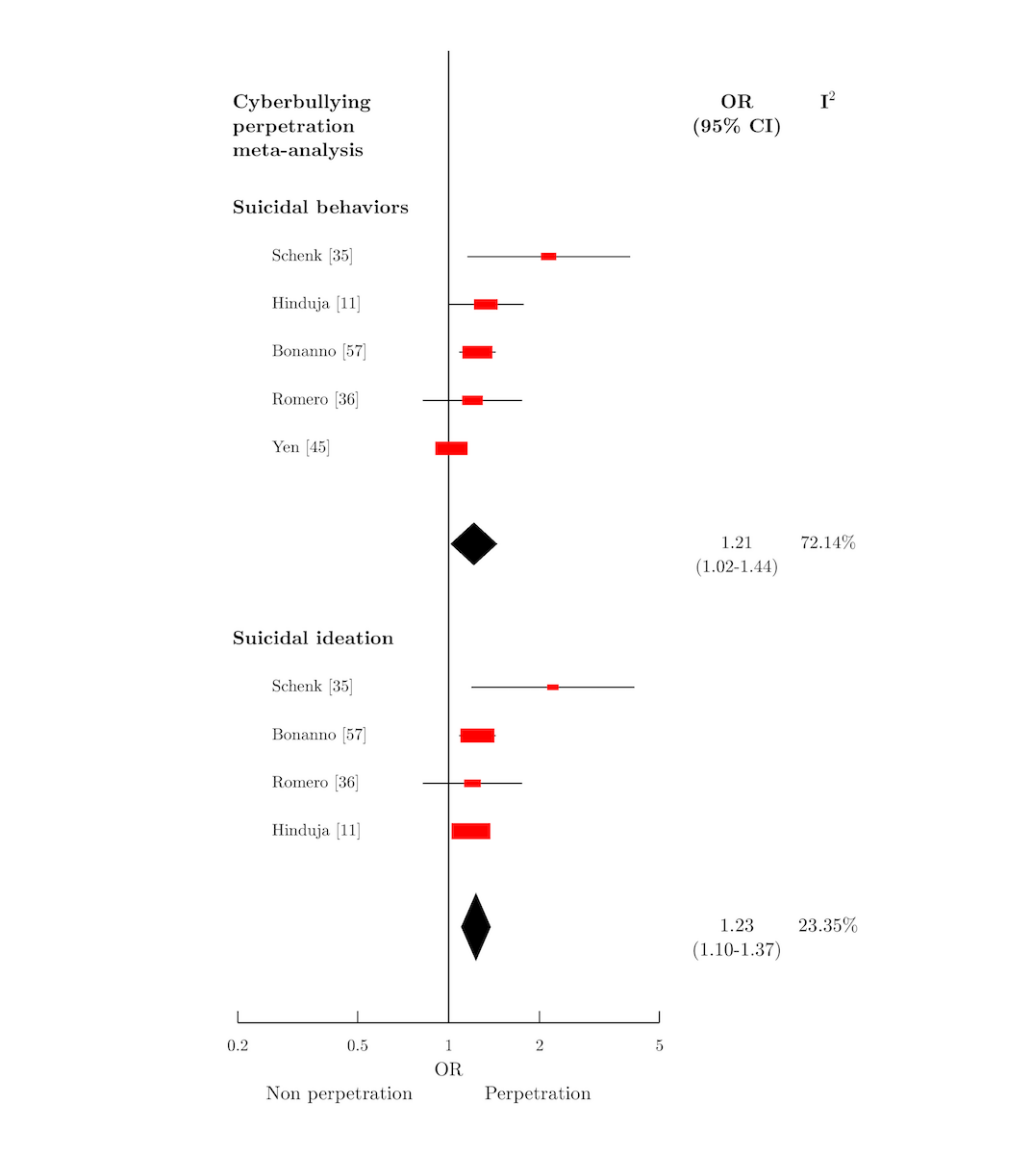
Forest plot of cyberbullying perpetration meta-analyses. OR: odds ratio.

Six meta-analyses were conducted (Table 1). Figure 2 displays the forest plots of the meta-analyses relating to cybervictimization, and Figure 3 displays those relating to cyberbullying perpetration. Further details of the measures included in all meta-analyses are available in Multimedia Appendix 4.

**Table 1:** Results of all meta-analyses performed. OR: odds ratio.

Cyberbullying group	Measure	*k*	n	OR (95 % CI)	ln OR (95 % CI)	*z*	*P*_z_	*T* ^2^	*Q*	*P*_Q_	I^2^ (%)
Victimization	Self-harm	11	85,967	2.35 (1.65-3.34)	0.85 (0.50-1.21)	4.76	<.001	0.30	174.92	<.001	94.28
Victimization	Suicidal behaviors	21	116,616	2.10 (1.73-2.55)	0.74 (0.55-0.94)	7.45	<.001	0.15	256.94	<.001	92.22
Victimization	Suicide attempt	10	85,541	2.57 (1.69-3.90)	0.94 (0.52-1.36)	4.41	<.001	0.39	171.48	<.001	94.75
Victimization	Suicidal ideation	16	103,774	2·15 (1.70-2.71)	0.76 (0.53-1.00)	6.39	<.001	0.17	157.62	<.001	90.48
Perpetration	Suicidal behaviors	5	4062	1.21 (1.02-1.44)	0.19 (0.02-0.37)	2.19	.03	0.02	14.36	.006	72.14
Perpetration	Suicidal ideation	4	3811	1.23 (1.10-1.37)	0.21 (0.10-0.32)	3.75	<.001	0.00	3.91	.27	23.35

#### Cybervictimization and Self-Harm

A total of 11 independent studies [11,16,18,20,42,48,50,54,55] (n=85,967) were eligible for meta-analysis of the association between cybervictimization and SH (Figure 2). A total of 7 articles were rated high quality, and 4 were rated medium. The meta-analysis produced OR 2.35 (95% CI 1.65-3.34).

#### Cybervictimization and Suicidal Behaviors

An empirical association between cybervictimization and suicidal behaviors was identified in 32 articles. Of these, 11 were of high quality, 16 medium, and 5 low. Regression coefficients ranged from beta=.15 (*P*<.01) for suicide risk [50] to beta=.97 (*P*<.001) for suicidal behavior [51]. ORs ranged from 1.73 (95% CI 1.26-2.38) for suicide attempt [54] to 6.32 (95% CI 1.44-8.69) for suicidal ideation [47]. Schenk et al [22] (medium quality) applied a χ^2^ goodness-of-fit producing χ^2^_2138_=9.1 (*P*=.03) when the frequencies of suicidal planning and attempts between cyber victims and controls were compared. Five papers found no significant association between cybervictimization and measures of suicidal behaviors [19,21,36,52,56].

A total of 21 studies [11,16-18,20,22,34,38,39, 42,45-52,54,55,57], with 116,616 participants, were included in the meta-analysis (Figure 2). Of these, 9 studies were rated high quality, 11 medium, and 1 low [38]. A number of studies were excluded from meta-analysis as a subsample of another study or for being ineligible [19,21,35-37,40,41,43,44,53,56]. The meta-analysis produced OR 2.10 (95% CI 1.73-2.55).

#### Cybervictimization and Suicide Attempt

A total of 10 studies [11,17,18,20,42,48-50,54,55] with 85,541 participants were eligible for inclusion in meta-analysis for this association (Figure 2). Of these, 7 studies were rated high quality and 3 as medium quality. The summary effect size of the association between cybervictimization and suicide attempt was OR 2.57 (95% CI 1.69-3.90).

#### Cybervictimization and Suicidal Ideation

A total of 16 studies [11,16,17,22,34,38,39,42,46,47,49] with 103,774 participants were included in the meta-analysis for this association (Figure 2). Of these, 7 studies were rated high quality, 7 medium quality, and 2 low quality. The summary effect size for this meta-analysis was OR 2.15 (95% CI 1.70-2.71).

#### Cyberbullying Perpetration and Suicidal Behaviors

The association between cyberbullying perpetration and suicidal behaviors was examined in 6 papers [11,18,35,36,45,57] (5 independent studies [11,35,36,45,57] with 4062 participants). Of the 5 studies included in the meta-analysis, 1 study was rated high quality, 3 medium, and 1 low. Combination of effect sizes was again applied where appropriate. The summary effect size for this association was OR 1.21 (95% CI 1.02-1.44).

#### Cyberbullying Perpetration and Suicide Attempt

Three articles [11,18,36] examined this association. One [11] found an OR of 1.49 (*P*<.05). Bauman et al [18] reported a direct effect of beta=.14 (*P*<.05) for males only, whereas a study based on a subsample of its population [36] found no significant effect. Meta-analysis was not conducted for the association between cyberbullying perpetration and suicide attempt as only 2 studies would be included.

#### Cyberbullying Perpetration and Suicidal Ideation

A total of 4 studies [11,35,36,57], with 3811 participants, were included in this meta-analysis. Of these, 1 was rated high quality, 2 medium, and 1 low. A summary effect size of OR 1.23 (95% CI 1.10-1.37) was produced for this association.

Heterogeneity between studies was both high and statistically significant in all cybervictimization meta-analyses. Heterogeneity was moderate and significant for the association between cyberbullying perpetration and suicidal behaviors, but nonsignificant for that of suicidal ideation. All calculated values of I^2^ are displayed in Table 1.

### Further Analyses

Two meta-regressions were performed: the first, for prevalence of traditional victimization against effect size for cybervictimization and suicidal behaviors, returned a standardized coefficient of beta=−.84. The second was for prevalence of traditional victimization against effect size for cybervictimization and suicidal ideation, producing a coefficient of beta=−.89. Both results were significant to the *P*<.001 level. This means that with increasing prevalence of traditional victimization comes a decrease in study effect size for the association between cybervictimization and suicidal behaviors, as well as that of cybervictimization and suicidal ideation.

A sensitivity analysis was conducted based solely on articles using a school-based sample. When compared with the results of the original meta-analyses, no significant difference was observed. We also conducted a sensitivity analysis based on articles that reported cybervictimization and traditional victimization separately, or which controlled for traditional victimization in their analyses. For each of the four cybervictimization meta-analyses, this produced greater ORs than those of the original meta-analyses, for example, SH produced OR 3.09 (95% CI 2.36-4.04) compared with OR 2.35 (95% CI 1.65-3.34), whereas suicidal behaviors produced OR 2.35 (95% CI 1.56-3.54) compared with OR 2.10 (95% CI 1.73-2.55). Fewer articles were included in each meta-analyses when restricted in this way (*k*=5 for SH, *k*=8 for suicidal behaviors, *k*=5 for suicide attempt, and *k*=6 for suicidal ideation).

Funnel plots were created for the cybervictimization and suicidal behaviors meta-analysis and cyberbullying perpetration and suicidal behaviors meta-analysis. These displayed no clear signs of publication bias.

More details regarding the results of the meta-regressions and sensitivity analyses, as well as figures for the funnel plots are available in Multimedia Appendix 4.

## Discussion

### Principal Findings

A total of 26 independent studies (33 articles) were included in this review covering a population of 156,384 children and young people younger than 25 years. A total of 20 independent studies (25 articles) found positive associations (negative influences) between cyberbullying victimization and SH or suicidal behaviors, or between cyberbullying perpetration and suicidal behaviors. One article [33] found an association (negative influence) between cybervictimization and unnatural child death (which included suicide). Two further articles [40,53], where the cyberbullying, SH, or suicidal behaviors measures had been combined with other measures, found negative influences. No significant associations were reported in 5 articles [19,21,36,52,56]. No positive influences of cyberbullying involvement were reported.

These associations were quantified in 6 meta-analyses: those who have experienced cybervictimization are 2.35 times as likely to SH, 2.10 times as likely to exhibit suicidal behaviors, 2.57 times more likely to attempt suicide, and 2.15 times more likely to have suicidal thoughts than nonvictims. Cyberbullying perpetrators were 1.21 times more likely to exhibit suicidal behaviors and 1.23 times more likely to experience suicidal ideation than nonperpetrators.

These findings were comparable with those found for traditional victimization in previous studies. One meta-analysis [13] reported ORs of 2.23 (95% CI 2.10-2.37) for the association between traditional victimization and suicidal ideation and OR 2.55 (95% CI 1.95-3.34) for suicide attempt. Another recent meta-analysis [58] also reported elevated odds of suicidal ideation and suicide attempt for victims of traditional bullying, with ORs of 1.77 (95% CI 1.56-2.02) and OR 2.13 (95% CI 1.66-2.73), respectively.

Only 5 of our eligible articles [17,33,42,48,50] presented results for cyberbullying independently of traditional bullying, with the relative contributions of both types of bullying impossible to determine in the majority of cases because results for cyberbullying involvement did not preclude the simultaneous occurrence of traditional bullying. We performed sensitivity analyses based on those articles that reported cyberbullying or traditional victimization separately or those that controlled for traditional victimization. In each case, ORs were greater than those of the original related meta-analyses, this suggests that cybervictimization could have a greater effect on SH and suicidal behaviors than does traditional victimization. It should be noted, however, that these sensitivity analyses were based on significantly fewer articles. Other evidence was also found suggesting that the effect of cybervictimization on SH and suicidal behaviors acted independently of its co-occurrence with traditional bullying [33,49,50], as well as some evidence of a cumulative effect [17,42,44,56] on SH and suicidal behaviors for the two types of bullying, although this was not seen in all studies [50]. We performed a meta-regression that showed that effect size decreased for cybervictimization and suicidal behaviors as the prevalence of traditional victimization increased. This was also seen for effect sizes in cybervictimization and suicidal ideation. This suggests that cybervictimization has a large effect on its own, but is less important when there are higher levels of traditional victimization present. It is possible that cyberbullying, which enables perpetrators to remain anonymous, has changed and extended the characteristics of both victims and perpetrators. Individuals who, in the nonvirtual world, were unlikely to be victimized as they were able to respond in person may be more vulnerable online where perpetrators may not be identified and are possibly emboldened in a way that they would not be face-to-face. Two recent cross-sectional studies have tried to shed some light on this issue, with one [59] finding that only 1% of adolescents reported being pure cyber victims, whereas the other [60] found traditional bullying to be far more common than cyberbullying, which should be taken into account when interpreting our findings.

Twenty-five new articles were identified since the previous systematic reviews and meta-analyses [12-15]. Van Geel et al [13] found that cybervictimization was more strongly related to suicidal ideation (OR 3.12, 95% CI 2.40-4.05) than in our meta-analysis; however, we included 13 more studies. Across studies, the weighted cybervictimization prevalence was calculated as 12.6% (95% CI 12.4%-12.7%). This is lower than estimates reported in some individual studies [11] of between 15% and 35%. A recent review of cyberbullying highlighted that variability in reported prevalence across studies was dependent on time frames and frequency of cyberbullying used in questions [61].

### Limitations

All included studies were observational in design and prone to bias (eg, recall and ecological fallacy). No conclusions can be drawn regarding causality and temporality from cross sectional or case‑control studies. Indeed, the possibility of reverse causality (ie, that SH or suicidal behaviors influence cyberbullying involvement) is currently not accounted for in the literature because of these limitations in study design. However, such study designs are appropriate, as manipulation of the level of exposure to cyberbullying involvement would be unethical [12]. We were unable to calculate the prevalence of both cyberbullying perpetration, SH, and suicidal behaviors because of a lack of relevant information.

Definitions of *cyberbullying* varied across studies. Few authors conducted their analyses by frequency of cyberbullying involvement. There is a lack of agreement among researchers as to the exact concept being researched, that is, electronic bullying (email and texts) and online internet harassment, as well as levels of repetition required and intentions of perpetrators. This has been highlighted in a recent review of cyberbullying [61] as the reason why such a broad range of instruments are used to assess cyberbullying. Although these issues may reflect the changing nature of communication technology and its use by young people, it does have an impact on case ascertainment. Future studies should do more to clarify the type of bullying under consideration, including expanding on the different mediums and modes of cyberbullying. For example, by making it clear if texts, social media, or emails are received (medium) and whether these were sent or received by just one individual or by an entire group (mode). This would allow for more precise analysis of whether these aspects had differential effects on outcomes, enabling those working in bullying prevention programs to equip children and young people with more closely tailored strategies for dealing with cybervictimization. We excluded one study [62] that asked “Have you ever felt hurt by a message you have seen on the internet or on a mobile website?” as it did not necessarily imply that the individual had “felt hurt” more than once, nor that the message was directed toward them.

Validated questionnaires were rarely used to determine SH and suicidal behaviors. Often these were assessed through the response to a single question from self-report surveys. Even where a more detailed methodology was employed, SH and suicidal status were often dichotomized, with all levels of severity grouped together (attempts, plans, and thoughts) to create outcomes such as suicidality and suicidal behaviors [35,45,50,51]. This will have had an impact on effect sizes. In particular, we were unable to distinguish the severity of suicidal ideation. Similarly for the SH and suicidal behavior meta-analyses, where it was not possible to isolate a single measure from two or more eligible measures, we computed a combined average with corresponding variance [11,17,18,42,46,49,54,55]. Although the pragmatic approach we employed was in keeping with both methodological guidance [30] and the literature [35,45,50,51], we acknowledge it may have had an impact on effect sizes in these meta-analyses and may appear reductive. It should be noted however that this was done for only a small proportion of papers per meta-analysis. We also computed a combined average when included studies presented results for females and males separately [18]. Only 1 ecological study assessed deaths [33] and found that countries with higher rates of cybervictimization were more likely to have higher incidence of unnatural child death, which included suicide. However, causality cannot be inferred from this type of study design, and there may be many other influences on deaths, including suicide. Few articles employed any statistical strategies to reduce bias because of confounding factors. Only 4 articles [22,35,47,53] used matching or propensity scoring while conducting their analyses, and this should be taken into account while interpreting our findings.

Sex differences in SH and suicidal behaviors are well recognized [63], yet, only 6 papers examined this with regard to cyberbullying [18,34,39,41,43,56], with conflicting results. We were unable to allow for the effects of other confounders in our meta-analyses (eg, past history of mental disorders or traditional bullying or suicidal behaviors), as these were either not reported, or reported in a way that did not allow us to distinguish them. Similarly, current traditional bullying involvement or mental health issues were presented in such a way that we could not distinguish whether individuals were suffering from more than one outcome (eg, whether cyber victims were suffering from both SH and mental health problems) or often one or more type of bullying. That we were unable to fully account for these two important factors in our analysis should be considered as perhaps the biggest limitation of our review. However, this is a reflection of current literature that should be addressed in future, ideally longitudinally designed, studies.

Some studies were excluded from certain meta-analyses where it was not possible to calculate the standard error for the effect size given the data presented [18,56]. As expected, the random-effects model indicated that variation in effects between studies existed. The high degree of heterogeneity (I^2^) observed between studies may reflect the varying victimization populations (case definitions and ascertainment), settings, and methodologies used in the calculation of individual effect sizes. Sensitivity analysis was performed for articles in which school-based samples were used. This revealed no significant change in effect sizes or heterogeneity across any of the outcomes examined and suggests that there exists some unexplained variance other than setting in this instance.

Heterogeneity of studies was particularly high for articles examining cyberbullying perpetration, and the quality of research was lower than that of cybervictimization. Only 8 articles investigated cyberbullying perpetration, with just 1 rated as high quality. It should be noted that the high degree of heterogeneity between studies (for both cybervictimization and perpetration) is a major limitation of the review. We acknowledge that the interpretation of summary OR statistics can be problematic. ORs and the relative risk diverge only when there are large effects (a twofold or threefold increase in risk) for groups already at a large initial risk. However it should be noted that where this occurs the interpretation is the same: these are large effects. We have presented CIs with our summary measures to address this issue.

We used funnel plots to investigate whether there were any signs of publication bias in our review. These gave no indication of bias, though it must be noted that the plot for cyberbullying perpetration was based on a low number of articles (*k=*5). However, despite conducting an extensive search, we cannot rule out the effects of publication bias on our results, and we only included English language studies. We attempted to address this by the breadth of our search; we identified many new studies compared with previously published reviews.

### Implications

This study highlights the significant impact that cyberbullying involvement can have on children and young people. Cybervictimization is a risk factor for SH and suicidal behaviors as is, to a lesser extent, cyberbullying perpetration for suicidal behaviors and ideation. Cyberbullying involvement should be considered by policy makers who implement bullying prevention (in addition to traditional bullying) and safe internet use programs. School, family, and community programs that promote appropriate use of technology are important. Prevention of cyberbullying should be included in school antibullying policies, alongside broader concepts such as digital citizenship, online peer support for victims, how an electronic bystander might appropriately intervene, and more specific interventions such as how to contact mobile phone companies and internet service providers to block, educate, or identify users. Suicide prevention and intervention is essential within any comprehensive antibullying program and should incorporate a whole-school approach to include awareness raising and training for staff and pupils.

A strong link between being a cyber victim and a perpetrator was found in some studies [18,36,45] This duality can particularly put males at higher risk of depression and suicidal behaviors [18]. These vulnerabilities should be recognized at school so that cyberbullying behaviors are seen not as disciplinary issues but as an opportunity to support vulnerable young people. Antibullying programs and protocols should address the needs of both victims and perpetrators. School exclusion might contribute to an individual’s sense of isolation. The relationship between cyber victims and suicidal behaviors appears robust. It may be that the persistent and pervasive nature of cybervictimization may lead to feelings of hopelessness, which are associated with suicidal behaviors in adolescents [64]. Students who are cybervictimized are less likely to report and seek help than those victimized by more traditional means [65,66]. Therefore it is important for staff to encourage help-seeking in relation to this issue.

Clinicians working with children and young people and assessing mental health issues should routinely ask about experiences of cyberbullying. The impact of cyberbullying should be included in the training of child and adolescent mental health professionals. Children and young people involved in cyberbullying should be screened for common mental disorders and SH.

The quality of study design, methods, and reporting in future studies needs improvement. Only a third of included articles (11 of 33) were rated as high quality, with 17 rated medium and 5 rated low. Validated psychometric instruments should be used to assess the suicidal status of individuals wherever possible to increase reliability and the ability to make comparisons across populations. More detailed analysis of the medium of cyberbullying (eg, via phones or instant messaging) should be explored to investigate any differences in populations and impact. The ability to distinguish media would support the development of targeted prevention strategies.

Finally, researchers should investigate the mechanisms by which mental disorders such as anxiety and depression mediate the link between cyberbullying involvement and SH and suicide. One study [18] found that depression mediated the link between cybervictimization and suicide attempts for females only, whereas perpetration was a direct predictor of suicide attempts for males only. This suggests that gender specific strategies for prevention and intervention may be helpful. Further research exploring the mechanisms of these associations is required. For cyberbullying perpetrators, a statement intended as a joke with no harm intended, may have unforeseen consequences with resultant guilt [18]. This association could be explored more deeply in mixed-methods and qualitative studies to gain a deeper understanding. Our review included no such studies. Future studies could also collect information from parents, peers, and teachers.

It is important to identify protective factors for children and young people exposed to cyberbullying. Although some studies [49] explored the moderating effects of social support (family and peers) on cyberbullying and mental health problems, no studies explored this in relation to SH and suicidal behaviors. Other protective factors may include aspects of resilience, such as internal locus of control and self-esteem [11].

### Conclusions

In conclusion, our review suggests that cyber victims are at greater risk of both SH and suicidal behaviors and, to a lesser extent, perpetrators of cyberbullying are at greater risk of suicidal behaviors than those with no cyberbullying involvement. The evidence base in this field has grown rapidly, but it is clear that the quality of future studies needs improvement. This research area would benefit from a clear definition of cyberbullying, assessed in longitudinal studies using validated assessments of SH and suicidal behaviors. Cyberbullying type, frequency, and gender should be explored. This is important to support policymakers, teachers, parents, clinicians, and others working with young people to make informed decisions in the safeguarding of children and young people.

## Appendix

Multimedia Appendix 1 Inclusion and exclusion criteria.

Multimedia Appendix 2 Further details of methods.

Multimedia Appendix 3 Summary table displaying the study type, mean age, quality score, and results of studies included in the review.

Multimedia Appendix 4 Further details of results.

Multimedia Appendix 5 Summary of outcome measures used in studies included in review.

Multimedia Appendix 6 Cybervictimization weighted prevalence rates and prevalence rates by study.

## Abbreviations

OR: odds ratio
SH: self-harm
