# Supplementary material for: Self-Harm, Suicidal Behaviours, and Cyberbullying in Children and Young People: Systematic Review
Source: J Med Internet Res. 2018 Apr 19;20(4):e129. doi: 10.2196/jmir.9044 (PMC5934539; doi:10.2196/jmir.9044)
Supplement: Multimedia Appendix 1 [file jmir_v20i4e129_app1.pdf]

Multimedia Appendix 1: Inclusion and Exclusion Criteria

Decision	Details
Include for data extraction	Investigates cyberbullying involvement and self-harm or suicidal behaviours
	**AND**
	Reports empirical data in sample under age 25
Studies of relevance but without empirical data	Investigates cyberbullying involvement and self-harm or suicidal behaviours
	**AND**
	Discusses possible causes, methods of prevention, risk and protective factors, or theoretical models in sample under age 25 but does not include empirical data
Use as background literature	Pertains to cyberbullying involvement and self-harm or suicidal behaviours
	**AND**
	Provides useful information on the above
Exclude as irrelevant to the review	Pertains to cyberbullying involvement and self-harm or suicide but does not fulfil above criteria
	**OR**
	Does not pertain to cyberbullying involvement, self-harm or suicidal behaviours in adolescence/young adulthood
