# Supplementary material for: Self-Harm, Suicidal Behaviours, and Cyberbullying in Children and Young People: Systematic Review
Source: J Med Internet Res. 2018 Apr 19;20(4):e129. doi: 10.2196/jmir.9044 (PMC5934539; doi:10.2196/jmir.9044)
Supplement: Multimedia Appendix 2 [file jmir_v20i4e129_app2.pdf]

Multimedia Appendix 2: Further Details of Methods

METHODS

Search strategy

An electronic literature search was conducted for all studies published from 1st January 1996 to the 3rd of February 2017, in the English language. The databases searched were CINAHL, Cochrane Library, EMBASE (excluding Medline journals), HMIC, Library catalogue and knowledge base, MEDLINE, NICE, PROSPERO, PsycINFO, PubMed, and SCOPUS. Additional searches were conducted in health improvement sources (Bibliomap, Database of Promoting Health Effectiveness Reviews, Health Evidence Canada) and topic-specific websites (American Association of Suicidology, Centre for Mental Health, National Health Service Scotland, Royal College of Psychiatrists) and meta-search engines (Google/Google Scholar). Grey literature was further explored by contacting experts in the field for any unreported or ongoing studies.

The following terms were searched in free text/keywords:

'Automutilation ', 'Distress*', 'Emotion*', 'nssi', '((oneself or myself or self) adj2 (cut* or harm* or hurt* or kill or injur* or mutilat*))', '(psychological adj (stress or distress))', 'SIB', 'Suicid*', 'Aol', 'Askfm', 'Bebo', 'blog*', 'chat room* OR chatroom*', 'cyber*', 'discussion forum', 'e-communi*','e-material*', 'Facebook', 'google*', 'hashtag', 'image sharing', 'Instagram', 'instant messag*', 'Internet*', 'live chat', 'live journal*', 'meme', 'MSN', 'Myspace', 'on line OR online', 'photo sharing', 'Pinterest', 'podcast*', 'social network*', 'spam*', 'troll*', 'Tumblr', 'tweet*', 'Twitter', 'video sharing', 'vine', 'virtual*', 'vlog*','web*','YouTube'

alongside the following database subject headings:

MESH: ‘Self-Injurious Behavior’, ‘Stress, Psychological’, ‘Blogging’, ‘Electronic Mail’, ‘Internet’, ‘Social media’, ‘Social networking’, ‘Bullying’, ‘Internet’, ‘Adolescent’, ‘Child’, ‘Students’, ‘Young adult’
HMIC: ‘Attempted suicide’, ‘Self harm’, ‘Suicide pacts’, ‘Suicide’, ‘Bullying’, ‘Cyberspace’, ‘Internet’, ‘internet websites’, ‘intranet’, ‘world wide web’
PsycInfo: ‘attempted suicide’, ‘self destructive behaviour’, ‘self injurious behavior’, ‘suicidal ideation’, ‘suicide prevention’, ‘cyberbullying’,‘ ‘Adolescent Attitudes’
EMBASE:‘automutilation’, ‘suicidal behaviour’, ‘suicide’, , ‘bullying’, ‘internet’, ‘social network’, ‘Adolescent’, ‘child’, ‘young adult’

Choice of effect size index and calculation

The common effect size index, the log odds ratio, was used in the meta-analysis. Other types of effect sizes were transformed into log odds ratios before the analysis. Inclusion criteria for the effect size index were based on the recommendations of Borenstein et al. [30]. Re-analyses of raw data or conversions were performed only when necessary. Studies that did not include measures of precision, i.e. confidence interval (CI) or *P*-value, were excluded from meta-analysis since these are required to calculate corresponding variances [31]. CIs were chosen in preference to *P*-values where possible. Where a paper only presented the maximum bound of a *P*-value, the true *P*-value was approximated in order to avoid biasing estimates. Papers that presented only the minimum bound of a *P*-value were excluded from the meta-analysis on the grounds of insufficient data.

Where a study presented more than one effect size eligible for a meta-analysis, the most appropriate measure to maintain homogeneity of outcomes was included, e.g. “suicide attempt” was chosen over “suicide attempt requiring medical treatment”. However, where it was not possible to make such a distinction, the effect sizes were

combined as an average, and a new corresponding variance computed taking account of the correlation between these outcomes [30]. If an article presented results in such a way that it was not possible to disaggregate the outcomes of interest from any other measures not considered in this review, then the article was rejected. Where two or more articles based on the same study population were eligible for meta-analysis, the article with greatest sample size was included. Underestimation of this correlation can lead to underestimation of the variance and overestimation of the precision. Therefore, where no data were presented on the correlation between the outcomes being combined, a correlation of $r = 1$ was assumed in order to avoid overestimation of the precision and biasing the result. Where results for both adjusted and unadjusted measures were presented (for confounding variables such as age, gender, and ethnicity), unadjusted results were used as per Borenstein et al. [30].

Meta-analysis was performed using MATLAB R2015a. The DerSimonian and Laird random-effects model was employed. Forest plots, summary effect sizes, CIs, *P*-values, and measures of heterogeneity in the form of the *Q*- and I^2-statistics were calculated. The I^2-statistic, as a proportion of inter-study dispersion, was interpreted as per the classification introduced by Higgins et al. [32]: low ($25\% \leq I^2 < 50\%$), moderate ($50\% \leq I^2 < 75\%$), and high ($I^2 \geq 75\%$).

To investigate the effect of traditional victimisation on study effect size, meta-regression was conducted for studies which reported a prevalence rate for traditional victimisation. Weighted least squares regression was used, with prevalence of traditional victimisation as covariate and study effect size (lnOR) as response variable. Standardised and unstandardised coefficients were computed as a measure of impact.

Two sensitivity analyses were performed for cybervictimisation. The first assessed the impact of study setting. This was done for articles whose samples were taken from school-based settings only. The second was based on all articles which analysed cybervictimisation separately of traditional victimisation or controlled for traditional victimisation. Results for both sensitivity analyses were then compared for differences with those of the original meta-analyses.

Finally, funnel plots were produced to investigate the possibility of publication bias. Since there was overlap in which articles appeared in the six meta-analyses, only two plots were produced: one for cybervictimisation and one for cyberbullying perpetration. In each case, the plot was based on the meta-analysis with the most number of articles.
