# Supplementary material for: Self-Harm, Suicidal Behaviours, and Cyberbullying in Children and Young People: Systematic Review
Source: J Med Internet Res. 2018 Apr 19;20(4):e129. doi: 10.2196/jmir.9044 (PMC5934539; doi:10.2196/jmir.9044)
Supplement: Multimedia Appendix 3 [file jmir_v20i4e129_app3.pdf]

Multimedia Appendix 3: Summary Table Displaying the Study Type, Mean Age, Quality Score, and Results of Studies Included in the Review.

Author, year, country	Study type, mean age, quality influence	Aim	Setting, n	Data collection method, response rate (RR)	Results
Alavi [37][a], 2015, Canada	Cross-sectional, 14.4, low negative	To examine the prevalence of bullying victimization (including cybervictimization) among adolescents referred for urgent psychiatric consultation, as well as the association between different types of bullying and suicidality.	Clinical, 375	Retrospective review of patient medical records, not applicable	Cyber victims were found to have experienced suicidal ideation more than nonvictims with odds ratio (OR)=3.60 (95% CI 1.30-10.90). Furthermore, those who had experienced cybervictimization reported more suicidal ideation than those who were verbally bullied (χ^2=4.1, df=1, *P*=.04). However, there was no statistically significant difference between those who had been both physically and verbally bullied and those who had been cybervictimized (χ^2=2.3, df=2, *P*=.12).
Arat [46][i,k], 2015, United States	Cross-sectional, 13.4, medium negative	To explore the relationship between various risk and protective factors (such as cyberbullying and school bullying) and psychosocial distress (suicidal ideation	School, 10,563	Self-report survey, RR=77%	Cyberbullying was found to have a significant effect with regard to suicidal ideation for each of the Asian, African, and Caucasian ethnic groups, with ORs of 3.54 (95% CI 2.12-5.93), 2.88 (2.47-3.34), and 2.59 (2.19-3.07), respectively.

		and depression) in students of three ethnic groups (Asian, African, and Caucasian).			However, only the Asian group produced a significant effect for cyberbullying and depression with OR 3.32 (2.07-5.30). With regard to the associations of school bullying, there were significant results for both the African and Caucasian groups with suicidal ideation, with ORs of 3.76 (2.99-4.74)and 4.53 (3.94-5.21), respectively, but none for the Asian group. Furthermore, all three ethnic groups reported significant effects for school bullying and depression with ORs of 1.53 (1.05-2.24], 3.32 (2.96-3.2), and 2.40 (2.04-2.83) for the Asian, African, and Caucasian groups, respectively.
Bannink [34][i,k], 2014, the Netherlands	Longitudinal study or cohort, 12.5, medium negative	To examine associations between traditional and cyber victimization with mental health problems and suicidal ideation.	School, 8271 (baseline); 3181 (follow-up)	Self-report survey, RR=95% (baseline); 38% (follow-up)	Cyber victims were significantly at risk of suicidal ideation compared with nonvictims, OR=1.74 (1.17-2.61), although this effect disappeared after controlling for baseline suicidal ideation OR=1.56 (0.80-1.87). Authors suggest his may be because of exposure time or small numbers. Gender was not found to have a significant interaction. Only female victims of cyberbullying were observed to be at a significant risk of mental health problems with OR=2.38 (1.45-3.91).
Bauman [18][h,i,j], 2013, United States	Cross-sectional survey, not given,	To study the associations among depression, suicidal behaviors, and bullying	School, 1491	Self-report survey, RR > 60%	Individuals were assessed by gender. No significant findings regarding the direct effect of cybervictimization and suicide attempts in females or males. When

	medium negative	and victimization in high school students.			including depression as a mediator, however, there was a significant indirect effect of beta=.23 (95% CI 0.13-0.33) in females, but not in males. Cyberbullying perpetration was found to be a significant predictor of suicide attempts in males with a direct effect of beta=.14 (*P*<.05), but not in females. When indirect effects mediated by depression were considered, no significant association was found in females (beta=−.18 [95% CI −0.13 to 0.11]) or males (beta=.00 [95% CI −0.09 to 0.08]). It should be noted of the former result, however, that the reported CIs do not encompass the effect itself.
Bonanno [57][i,k,l,m], 2013, Canada	Cross-sectional survey, 14.2, medium negative	To examine the association between involvement in cyberbullying and depressive symptomatology and suicidal ideation.	School, 399	Self-report survey, RR=100%	Victims of cyberbullying were at a greater risk of suicidal ideation (measured with a validated questionnaire) compared with nonvictims, beta=.25 (*P*<.001). Cybervictimization contributed to depressive symptomatology, with regression coefficient beta=.11 (*P*=.03). Cyberbullying perpetration was significantly associated with suicidal ideation, beta=.22 (*P*<.001) and depressive symptomatology beta=.16 (*P*<.05).

Cassidy [19], 2009, Canada	Cross-sectional, not given, low none	To report on a cyberbullying study conducted in five Canadian schools and obtain as much information as possible on participants' cyberbullying experiences.	School, 365	Self-report survey, RR=not given	3.8% of students said that they had received threatening messages from another student that had made them feel afraid and that this had induced in them suicidal thoughts.
Cénat [39][b,i,k], 2015, Canada	Cross-sectional, 15.4, high negative	To explore correlates between various forms of bullying (cyberbullying, homophobic bullying, and bullying at school or elsewhere) and psychological distress, low self-esteem, and suicidal ideation for sexual-minority youth.	School, 8194	Self-report survey, RR=99%	Cybervictimization was significantly associated with all three mental health indicators: suicidal ideation, beta=.63 (95% CI 0.47-0.78); psychological distress, beta=.64 (95% CI 0.50-0.79); and low self-esteem, beta=.36 (95% CI 0.24-0.49]. Additionally, both other forms of bullying were significantly associated with suicidal ideation: homophobic bullying with beta=.61 (95% CI 0.29-0.93) and bullying at school or elsewhere with beta=.40 (0.25-0.56).
DeSmet [47][i,k], 2014, Belgium	Case-control study, 15.3, medium negative	To assess the associations between both cybervictimization and traditional bullying in obese youth and psychosocial distress (suicidal ideation, self-esteem, and quality of life [QoL]) and barriers and facilitators to a healthy lifestyle.	Clinical, 204	Self-report survey, RR=not given	Cybervictimized obese youth were OR 6.32 (95% CI 1.44-8.69) times more likely to have experienced suicidal thoughts than nonvictimized obese youth. No significant association was found, however, between cybervictimization in obese youth and self-esteem, QoL, or barriers and facilitators of a healthy lifestyle.

Duong [48][h,i,j], 2014, United States	Cross-sectional, not given, medium negative	To investigate the relationship between both cyber- and school bullying in sexual minority youth (SMY) and aggressive and suicidal behaviors.	School, 951	Self-report survey, RR=83%	SMY who had been cybervictimized were OR 3.07 (95% CI 1.39-6.79) times as likely to have attempted suicide than nonvictimised SMY. However, no significant association was found between cybervictimization and serious suicide attempt. There was also an accumulative effect was for those who had experienced both cyber- and school bullying, with victims of this type being OR 5.10 (1.90-13.71) times as likely to have attempted suicide and OR 5.03 (95% CI 1.2-14.70) times as likely to have made a serious suicide attempt.
Elgar [49][h,i,j,k], 2014, United States	Cross-sectional, 15.0, high negative	To examine the unique association between cybervictimization and mental health and to explore the potential moderating role of family contact. Internalizing problems were also measured, which included self-harm and suicide attempts in the previous 12 months.	School, 18,834	Self-report survey, RR > 90%	Suicidal thoughts, n=2364; self-harm, n=2172; suicide attempts, n=911. Cybervictimization was categorized by frequency ("rarely," "sometimes," and "often") with victims being found to be at an elevated risk across all outcomes when compared with nonvictims. In particular, there were ORs for self-harm of 1.99, 2.66, and 3.30, respectively. For suicide attempt these were 1.81, 3.01, and 3.47, respectively, whereas for suicidal thoughts these were 1.84, 2.43, and 2.97, respectively. Cyberbullying perpetration was not analyzed.
Fu [33], 2014, 24 European countries	Ecological, not given, medium negative	To explore the ecological relationship between Internet risk exposure and unnatural child death.	Participants' home, 25,142	Face-to-face researcher-led survey, RR=not given	Children aged 10 to 14 years**.** Four Internet-based risks were identified that included both traditional and online bullying, exclusively online bullying,

					Internet addiction, and exposure to online information on self-harm and suicide. Quasi-Poisson regression was conducted on the prevalence of these exposures with unnatural child deaths. Statistically significant associations were only found for traditional and online bullying and exclusively online bullying, with regression coefficients of beta=.12 and .24, respectively. A 1% rise in the prevalence of cybervictimization translated into a 28% increase in risk of unnatural child death.
Goebert [20][h,i,j], 2011, United States	Cross-sectional, not given, medium negative	To investigate the relationship between cybervictimization and mental health problems, as well as suicidality, in a multi-ethnic sample of students from two high schools in Hawai'i, United States.	School, 677	Self-report survey, RR=33%	Those who had been cybervictimized were found to be OR 3.22 (95% CI 1.82-5.70) times more likely to have attempted suicide than those who had not. There was also a statistically significant relationship between cybervictimization and depression, with OR=1.85 (95% CI 1.30-2.63) but not for anxiety. Chi-squared tests were also run for cybervictimization to examine potential group differences in gender and ethnicity; however, no significant effect was detected for either variable.
Hay [16][c,h,i,k], 2010a, United States	Cross-sectional, 15.0, medium negative	To test three separate hypotheses on the effects of cybervictimization and traditional victimization on self-harm and suicidal ideation.	School, 426	Self-report survey, RR=93%	Cybervictimization was found to be significantly associated with both self-harm and suicidal ideation, with regression coefficients of beta=.38 (*P*<.001) and beta=.39 (*P*<.001), respectively. Traditional victimization

					was also associated with these outcomes, giving coefficients of beta=.32 (*P*<.001) and beta=.39 (*P*<.001), respectively.
Hay [41][c], 2010b, United States	Cross-sectional, 15.0, medium negative	To examine the effects of both cyber- and traditional bullying on the internalizing behaviors of self-harm and suicide, as well as other various externalizing behaviors.	School, 424	Self-report survey, RR=93%	Cybervictimization had an effects of beta=.39 for self-harm, beta=.41 for suicidal ideation, and beta=.33 for the externalizing behavior of delinquency. These effects were all slightly higher than that of the corresponding effects for traditional bullying, which were beta=.33, beta=.39, and beta=.22 for the above named measures, respectively. All effects were significant to the *P*<.01 level
Hébert [40], 2016, Canada	Cross-sectional, not given, high negative	To explore the association between cybervictimization, traditional victimization, and history of child sexual abuse and mental health problems, which was measured as a combination of self-esteem, psychological distress, and suicidal ideation.	School, 14,974	Self-report survey, RR=99%	Cybervictimization was found to have a direct effect of beta=.30, whereas traditional victimization and history of child sexual abuse had direct effects of beta=.40 and beta=.70, respectively. All results were significant to the *P*<.05 level.
Hinduja [11][h,i,j,k,l,m], 2010, United States	Cross-sectional, 12.8, high negative	To examine how cyberbullying and traditional bullying, as victim or perpetrator, affects suicidal ideation and suicide attempt in a random sample of 1963	School, 1963	Self-report survey, RR=96%	For suicidal ideation, there were significant associations with cybervictimization (beta=.25), cyberbullying perpetration (beta=.17), traditional victimization (beta=.23), and traditional perpetration (beta=.16). Associations with suicide attempt were

		middle-school students from one of the largest school districts in the United States.			also found for cybervictimization (OR=1.94), cyberbullying perpetration (OR=1.49), traditional victimization (OR=1.68), and traditional perpetration (OR=2.08). Forms of cyberbullying occurred most often via email (18.3%), instant messaging (16.0%), MySpace (14.2%), and chat rooms (10.0%).
Kindrick [44][d], 2013, United States	Cross-sectional, not given, medium negative	To use a sample of high school students to study the relationship between school bullying or cyberbullying and missing school because of feeling unsafe, depression, and suicide.	School, 1375	Self-report survey, RR=not given	Being a victim of cyberbullying, school bullying, or both increased the risk for suicidal ideation (OR=4.50, 2.80, and 6.40, respectively), suicide plan (OR=3.50, 2.10, and 4.60, respectively), and depression (OR=3.60, 2.80, and 4.60, respectively), but did not have statistically significant associations with the measures for suicide attempt or treated for suicide attempt. Furthermore, “feeling unsafe at school” was a significant risk factor for suicidal ideation (OR=2.30), suicide plan (OR=2.40), suicide attempt OR=3.90, treated for suicide attempt (OR=5.10), and depression (OR=3.00).
Kodish [50][h,i,j], 2016, United States	Cross-sectional, 16.8, high negative	To explore whether experiences with cyber-, physical, and verbal bullying (as well as the cumulative effect of these types of bullying) were uniquely associated with	Clinical, 5429	Self-report survey, RR=not given	All forms of bullying victimization were found to be significantly associated with elevated suicide risk, with regression coefficients of beta=.15, beta=.16, and beta=.15 cyber-, physical, and verbal bullying, respectively, with cumulative bullying effect of beta=.12, all significant to the *P*<.01 level. Cybervictimization

		suicide risk and suicide attempt.			and verbal bullying were also found to be associated with suicide attempt with coefficients of beta=.73 and beta=.87, respectively, both also significant to the *P*<.01 level. However, physical bullying and cumulative bullying did not have significant effects for this relationship.
Litwiller [51][i], 2013, United States	Cross-sectional, 16.1, high negative	To examine the relationship between victimization from both physical bullying and cyberbullying and adolescent suicidal behaviors, while also testing violent behavior, substance use, and sexual behavior as mediators for this in 27 schools over 7 countries.	School, 4693	Self-report survey, RR=65%	Cybervictimization was found to have a direct effect of beta=.97 (*P*<.001) and a total indirect effect of OR 0.48 (95% CI 0.45-0.51) on suicidal behaviors using multiple mediation analysis. This was partially mediated by substance use and violent behavior that had specific indirect effects of OR 0.32 (95% CI 0.28-0.35) and OR 0.16 (95% CI 0.14-0.19), respectively. Sexual behavior was not a significant mediator between cybervictimization and suicidal behaviors.
Messias [42][e,h,i,j,k], 2014, United States	Cross-sectional, not given, high negative	To study the relationship between school and cyberbullying involvement and depression and suicidality among teens.	School, 15,425	Self-report survey, RR=87%	The outcomes included four suicidality measures and a 2-week sadness measure, which were all analyzed against cybervictimization, school victimization, or both. Cyber victims were found to have a greater risk than nonvictims of suicidal ideation, suicide plan, suicide attempt, suicide attempt requiring treatment, and 2-week sadness with ORs of 3.40, 3.10, 3.60, 4.00, and 370, respectively. School bullying victims were also at increased risk than were

					nonvictims, with ORs for these outcomes of 2.60, 2.60, 2.20, 1.60, and 2.20, respectively. Those at highest risk were both victims of cyberbullying and school bullying. When compared with nonvictims, ORs for the above outcomes were 5.50, 5.30, 5.50, 4.40, and 5.70.
Mitchell [52][i,k], 2014, United States	Cross-sectional, 14.5, medium none	To examine whether exposure to websites that encourage self-harm or suicide is related to thoughts of self-harm or suicide in the past 30 days.	Participants' home, 1560	Researcher-led telephone interview, RR=65%	There were no significant findings regarding online harassment as a predictor for thoughts of self-harm or thoughts of suicide. The only risk factors that showed any significant association with thoughts of self-harm were self-harm or suicide website usage (OR 11.20 [95% CI 3.70-33.70]), depression symptoms (OR 1.50 [95% CI 1.30-1.60]), and dating-violence victim (OR 3.80 [95% CI 1.10-13.30]).
Price [21], 2010, Australia	Cross-sectional, not given, low none	Seek to extend knowledge around the use and perceived effectiveness of coping strategies for cyberbullying, as defined by young people themselves, as well as the prevalence and impact of cyberbullying.	Online, 548	Self-report survey, RR=not given	3% of participants, all of whom had experienced cybervictimization, indicated through free-text answers that they had experienced suicidal thoughts as a result of cybervictimization. A further 2% indicated that they had self-harmed because of cybervictimization.
Reed [43][e], 2015, United States	Cross-sectional, 16.1, high negative	To explore the relationships between cyber- and traditional bullying and measures of suicidality (suicidal	School, 15,425	Self-report survey, RR=81%	A path model suggested that the relationships between cybervictimization and traditional bullying and suicidal thinking were mediated by both depression and substance abuse.

		ideation, suicide planning, and suicide attempt), as well as how other factors such as depression and substance abuse might mediate the link between these.			Furthermore, it suggested that both cybervictimization and traditional victimization and suicide attempt were mediated by violent behavior. There were also unstandardized total effects (and 99.9% CIs) for the association between cybervictimiztion and suicidal thinking, suicide planning, suicide attempt, and depression of OR 0.64 (95% CI 0.38-0.84), OR 0.75 (95% CI 0.43-1.07), OR 0.85 (95% CI 0.46-1.24), and OR 0.45 (95% CI 0.31-0.65).
Roberts [38][i,k], 2016, Canada	Cross-sectional, 14.5, low negative	To study the prevalence of cybervictimization and traditional bullying among adolescents referred for psychiatric emergency assessment, as well as its relationship with suicidal ideation.	Clinical, 805	Retrospective review of patient medical records, RR=not given	Patients who had reported being cybervictimized had OR of 4.39 (95% CI 2.24-8.62) compared with nonictims for suicidal ideation. No significant effect, however, was found for this measure in those who had been traditionally bullied. Cyber victims and traditional victims were also compared directly, with the former being OR 2.56 (95% CI 1.28-5.12) times more likely to have experienced suicidal ideation.
Roh [53], 2015, South Korea	Cross-sectional, 14.3, medium negative	To investigate the relationships of different classes of cooccurring bullying and suicidal behaviors, including suicidal ideation and suicide attempt.	Clinical, 4410	Researcher-led interview and surveys, RR=not given	Bullying experiences were split into two classes: physical bullying and nonphysical bullying (which included cyberbullying). Those in the nonphysical bullying class were found to be 2.24 (P<.01) times as likely to have experienced suicidal ideation as those who had not experienced any form of bullying and were also 3.28 (P<.001)

					times more likely to have attempted suicide.
Romero [36][f,k,l,m], 2013, United States	Cross-sectional, not given, low none	To investigate suicide attempts, suicidal ideation, and suicide plan among adolescent Latina girls, with a focus on traditional and cyberbullying as predictors (victimization and perpetration in both cases).	School, 650	Self-report survey, RR=not given	There were no statistically significant findings with regard to cyberbullying involvement as victim or perpetrator for suicidal ideation, suicide attempt, or suicide plan. Cybervictimization, ORs 1.10, 1.10, and 0.89 (suicide attempt, suicidal ideation, and suicide plan, respectively). Cyberbullying perpetration ORs of 1.20, 1.20, and 1.42 for the same measures.
Sampasa-Kanyinga [54][h,i,j,k], 2014, Canada	Cross-sectional, 14.3, high negative	To explore the associations between cyberbullying and school bullying and suicidal ideation, suicide planning, and suicide attempt in middle and high school students.	School, 2999	Self-report survey, RR=68%	Both forms of bullying investigated were found to have statistically significant relationships with the three measures of suicidality: suicidal ideation, suicide planning, and suicide attempt. For cyberbullying these were ORs of 3.31 (95% CI 2.16-5.07), 2.79 (95% CI 1.63-4.77), and 1.73 (95% CI 1.26-2.38) when compared with nonvictims, respectively, whereas for school bullying, these were ORs of 3.48 (95% CI 2.48-4.89), 2.76 (95% CI 2.20-3.45), and 1.64 (95% CI 1.18-2.27), when compared with nonvictims, respectively.
Schenk [22][g,i,k], 2012, United States	Case-control study, 20.0, medium negative	To investigate prevalence, psychological impact, and coping strategy in college victims of cyberbullying in the United States.	Higher education institute, 799	Self-report survey, RR=not given	Results showed that there was a significant difference in the frequency of suicidal planning or attempts between victims of cyberbullying and controls with χ^2=9.1 (df=2138, *P*=.03) and a significant difference in the frequency of

					suicidal ideation between victims and controls with χ^2=7.4 (df=2138, *P*=.03). There was no significant effect for gender and suicidal behaviors. When compared with controls, cyber victims were also found to have an elevated risk of depression (*F*=9.90), anxiety (*F*=9.11), phobic anxiety (*F*=8.66), and paranoia (*F*=12.20). Although gender differences were also considered, no significant findings were found. All of the subscales of the Symptom Checklist-90-R (SCL-90-R) as a whole were analyzed between cyber victims and controls. This was shown to be statistically significant with *F*=3.35 (*P*=.001).
Schenk [35][g,l,m], 2013, United States	Case-control study, 20.0, medium negative	To investigate the psychological symptomatology, suicidal behaviors, aggressive tendencies, and illegal behaviors of university cyberbullying perpetrators.	Higher education institute, 799	Self-report survey, RR=not given	60 (7.5%) participants endorsed cyberbullying someone at least four times. 19 (2.4%) were both perpetrators and victims**.** Perpetrators and perpetrator or victims had a significantly higher likelihood of attempting suicide than controls with $F_{2,151}$=6.96 (*P*=.001), threatening suicide with $F_{2,150}$=4.57 (*P*=.012) and total Suicide Behaviors Questionnaire-Revised score with *F*=4.23 (*P*=.016). There was no significant difference, however, for suicide plan or suicidal ideation. Perpetrators and perpetrator or victims were also found to have significantly

					higher scores in interpersonal sensitivity (*F*=6.87), depression (*F*=7.92), hostility (*F*=7.94), phobic anxiety (*F*=5.51), paranoia (*F*=8.11), and psychosis (*F*=9.30), as well as in their total overall score on the SCL-90-R (*F*=1.87).
Schneider [17][h,i,j,k], 2012, United States	Cross-sectional, not given, high negative	To use data from a census of high school students to document the prevalence of cyberbullying and school bullying victimization and their associations with psychological distress.	School, 20,406	Self-report survey, RR=88%	Results indicated that cyber victims were more likely than nonvictims to suffer from self-injury (OR 3.56 [95% CI 2.95-4.29]), suicidal ideation (OR 3.35 [95% CI 2.71-4.13]), suicide attempt (OR 5.00 [95% CI 3.73-6.71]), suicide attempt requiring treatment (OR 5.36 [95% CI 3.28-8.75]), and depressive symptoms (OR 3.26 [95% CI 2.76-3.85]). School bullying victims were also found to have a higher chance of suffering from all of the above outcomes, with ORs of 2.49, 2.07, 2.11, 2.16, and 2.31, respectively. Finally, those who were victims of both cyberbullying and school bullying were at the highest risk compared with nonvictims, with ORs for the above outcomes of 6.52, 6.86, 8.64, 10.93, and 5.64, respectively.
Sinclair [55][h,i,j,k], 2012, United States	Cross-sectional, not given, high negative	To examine how two forms of interstudent harassment (cyber- and Lesbian, Gay, Bisexual, Transgender, and Queer bias-based harassment) are associated with academic,	School, 17,366	Self-report survey, RR=not given	It was found that those who had experienced nonbiased cybervictimization were at a greater risk of self-harm than those who had not (OR=2.95). This was also the case for victims of biased-based cyberbullying (OR=4.75). Suicidal ideation was also

		substance use, and mental health problems.			found to be more common among nonbiased cyber victims and biased-based cyber victims with ORs of 4.15 and 6.85, respectively. Furthermore, the chance of having attempted suicide was higher for nonbiased and biased-based cyber victims with ORs of 3.82 and 7.85, respectively. No CIs or *P* values reported.
Turner [56], 2013, United States	Cross-sectional, 13.8, medium none	To investigate whether type of bullying and gender of victim are important in terms of a victim's susceptibility with relation to depression and suicidal ideation.	School, 2523	Self-report survey, RR=74%	Victims of cyberbullying were not found to be significantly at risk of suicidal ideation when compared with nonvictims. This was also the case for victims of verbal bullying and physical bullying. When these three types were considered together as general bullying victimization, it was found that victims were more likely to experience suicidal ideation than nonvictims, with beta=.07. This was also observed in the female and male models (coefficients of .06 and .08, respectively). Cybervictimization in the full and male samples were not significantly associated with depression; however, it was found that female victims were more likely to suffer from depression than nonvictims with beta=.49. Many CIs presented in this paper were outside point estimates.
Yen [45][i,l], 2014, Taiwan	Cross-sectional, 13.1,	To examine the prevalence rates and multilevel correlates of cyber victims	Clinical records, 251	Researcher-led interview, clinical observations, and	Cyber victims were found to be at higher risk of suicidality than nonvictims with beta=.23 (*P*=.001) for this association.

	medium negative	and perpetrators among Taiwanese male adolescents diagnosed with attention-deficit hyperactivity disorder, as well as the relationships between cyberbullying involvement and depression, anxiety, and suicidality (combined measure of self-report attempts and ideation).		review of patient medical, RR=90%	Victims also had elevated risk of depression (beta=.22, *P*=.001) but no significant association with anxiety was found. The association between cyberbullying perpetration and the above outcomes was also analyzed; however, no significant association was found with suicidality, depression, or anxiety.

[a]Subsample of Roberts [38].

[b]Subsample of Hébert [40].

[c]Share the same population.

[d]Subsample of Messias [42] and Reed [43].

[e]Share the same population.

[f]Subsample of Bauman [18].

[g]Share the same population

[h]Included in cybervictimization and self-harm meta-analysis.

[i]Included in cybervictimization and suicidal behaviors meta-analysis.

[j]Included in cybervictimization and suicide attempt meta-analysis.

[k]Included in cybervictimization and suicidal ideation meta-analysis.

[l]Included in cyberbullying perpetration and suicidal behaviors meta-analysis.

[m]Included in cyberbullying perpetration and suicidal ideation meta-analysis.

References

11. Hinduja S, Patchin JW. Bullying, cyberbullying, and suicide. Arch Suicide Res 2010;14(3):206-21. PMID:20658375

16. Hay C, Meldrum R. Bullying victimization and adolescent self-harm: Testing hypotheses from general strain theory. J Youth Adolesc 2010a;39(5):446-59. PMID:20072852

17. Schneider SK, O'Donnell L, Stueve A, Coulter RW. Cyberbullying, school bullying, and psychological distress: A regional census of high school students. Am J Public Health 2012;102(1):171-7. PMID:22095343

18. Bauman S, Toomey RB, Walker JL. Associations among bullying, cyberbullying, and suicide in high school students. J Adolesc 2013;36(2):341-50. PMID:23332116

19. Cassidy W, Jackson M, Brown KN. Sticks and stones can break my bones, but how can pixels hurt me? Students' experiences with cyber-bullying. Sch Psychol Int 2009;30(4):383-402. doi:10.1177/0143034309106948

20. Goebert D, Else I, Matsu C, Chung-Do J, Chang JY. The impact of cyberbullying on substance use and mental health in a multiethnic sample. Matern Child Health J 2011;15(8):1282-6. PMID:20824318

21. Price M, Dalgleish J. Cyberbullying: Experiences, impacts and coping strategies as described by Australian young people. Youth Studies Australia 2010;29(2):51-9.

22. Schenk AM, Fremouw WJ. Prevalence, psychological impact, and coping of cyberbully victims among college students. J Sch Violence 2012;11(1):21-37. doi:10.1080/15388220.2011.630310

33. Fu K-w, Chan C-h, Ip P. Exploring the relationship between cyberbullying and unnatural child death: an ecological study of twenty-four European countries. BMC Pediatr 2014;14(1):195. PMID:25079144

34. Bannink R, Broeren S, van de Looij–Jansen PM, de Waart FG, Raat H. Cyber and traditional bullying victimization as a risk factor for mental health problems and suicidal ideation in adolescents. PLoS One 2014;9(4):e94026. PMID:24718563

35. Schenk AM, Fremouw WJ, Keelan CM. Characteristics of college cyberbullies. Comput Human Behav 2013;29(6):2320-7. doi:10.1016/j.chb.2013.05.013

36. Romero AJ, Wiggs CB, Valencia C, Bauman S. Latina teen suicide and bullying. Hisp J Behav Sci 2013;35(2):159-73. doi:10.1177/0739986312474237

37. Alavi N, Roberts N, Sutton C, Axas N, Repetti L. Bullying victimization (being bullied) among adolescents referred for urgent psychiatric consultation: prevalence and association with suicidality. Can J Psychiatry 2015;60(10):427-31. PMID:26720189

38. Roberts N, Axas N, Nesdole R, Repetti L. Pediatric Emergency Department Visits for Mental Health Crisis: Prevalence of Cyber-Bullying in Suicidal Youth. Child Adolesc Social Work J 2016;33(5):469-72. doi:10.1007/s10560-016-0442-8

39. Cénat JM, Blais M, Hébert M, Lavoie F, Guerrier M. Correlates of bullying in Quebec high school students: The vulnerability of sexual-minority youth. J Affect Disord 2015;183:315-21. PMID:26047959

40. Hébert M, Cénat JM, Blais M, Lavoie F, Guerrier M. Child sexual abuse, bullying, cyberbullying, and mental health problems among high schools students: a moderated mediated model. Depress Anxiety 2016;33(7):623-9. PMID:27037519

41. Hay C, Meldrum R, Mann K. Traditional bullying, cyber bullying, and deviance: A general strain theory approach. J Contemp Crim Justice 2010b;26(2):130-47. doi:10.1177/1043986209359557

42. Messias E, Kindrick K, Castro J. School bullying, cyberbullying, or both: Correlates of teen suicidality in the 2011 CDC youth risk behavior survey. Compr Psychiatry 2014;55(5):1063-8. PMID:24768228

43. Reed KP, Nugent W, Cooper RL. Testing a path model of relationships between gender, age, and bullying victimization and violent behavior, substance abuse, depression, suicidal ideation, and suicide attempts in adolescents. Child Youth Serv Rev 2015;55:128-37. doi:10.1016/j.childyouth.2015.05.016

44. Kindrick K, Castro J, Messias E. Sadness, suicide, and bullying in Arkansas: results from the Youth Risk Behavior Survey--2011. J Ark Med Soc 2013;110(5):90-1. PMID:24383197
45. Yen C-F, Chou W-J, Liu T-L, Ko C-H, Yang P, Hu H-F. Cyberbullying among male adolescents with attention-deficit/hyperactivity disorder: Prevalence, correlates, and association with poor mental health status. Res Dev Disabil 2014;35(12):3543-53. PMID:25241113
46. Arat G. Emerging protective and risk factors of mental health in Asian American students: findings from the 2013 Youth Risk Behavior Survey. Vulnerable Child Youth Stud 2015;10(3):192-205. doi:10.1080/17450128.2015.1045437
47. DeSmet A, Deforche B, Hublet A, Tanghe A, Stremersch E, De Bourdeaudhuij I. Traditional and cyberbullying victimization as correlates of psychosocial distress and barriers to a healthy lifestyle among severely obese adolescents – a matched case–control study on prevalence and results from a cross-sectional study. BMC Public Health 2014;14(1):224. PMID:24593118
48. Duong J, Bradshaw C. Associations between bullying and engaging in aggressive and suicidal behaviors among sexual minority youth: The moderating role of connectedness. J Sch Health 2014;84(10):636-45. PMID:25154527
49. Elgar FJ, Napoletano A, Saul G, Dirks MA, Craig W, Poteat VP, Holt M, Koenig BW. Cyberbullying victimization and mental health in adolescents and the moderating role of family dinners. JAMA Pediatr 2014;168(11):1015-22. PMID:25178884
50. Kodish T, Herres J, Shearer A, Atte T, Fein J, Diamond G. Bullying, depression, and suicide risk in a pediatric primary care sample. Crisis 2016. PMID:27040126
51. Litwiller BJ, Brausch AM. Cyber bullying and physical bullying in adolescent suicide: the role of violent behavior and substance use. J Youth Adolesc 2013;42(5):675-84. PMID:23381779
52. Mitchell KJ, Wells M, Priebe G, Ybarra ML. Exposure to websites that encourage self-harm and suicide: Prevalence rates and association with actual thoughts of self-harm and thoughts of suicide in the United States. J Adolesc 2014;37(8):1335-44. PMID:25313930
53. Roh B-R, Yoon Y, Kwon A, Oh S, Lee SI, Ha K, Shin YM, Song J, Park EJ, Yoo H. The structure of co-occurring bullying experiences and associations with suicidal behaviors in Korean adolescents. PLoS One 2015;10(11):e0143517. PMID:26619356
54. Sampasa-Kanyinga H, Roumeliotis P, Xu H. Associations between cyberbullying and school bullying victimization and suicidal ideation, plans and attempts among Canadian schoolchildren. PLoS One 2014;9(7):e102145. PMID:25076490
55. Sinclair KO, Bauman S, Poteat VP, Koenig B, Russell ST. Cyber and bias-based harassment: Associations with academic, substance use, and mental health problems. J Adolesc Health 2012;50(5):521-3. PMID:22525118
56. Turner MG, Exum ML, Brame R, Holt TJ. Bullying victimization and adolescent mental health: General and typological effects across sex. J Crim Justice 2013;41(1):53-9. doi:10.1016/j.jcrimjus.2012.12.005
57. Bonanno RA, Hymel S. Cyber bullying and internalizing difficulties: Above and beyond the impact of traditional forms of bullying. J Youth Adolesc 2013;42(5):685-97. PMID:23512485
