# Supplementary material for: Self-Harm, Suicidal Behaviours, and Cyberbullying in Children and Young People: Systematic Review
Source: J Med Internet Res. 2018 Apr 19;20(4):e129. doi: 10.2196/jmir.9044 (PMC5934539; doi:10.2196/jmir.9044)

Multimedia Appendix 4: Further Details of Results

Study populations

Twenty articles were based on independent studies and unique populations. Six sets of articles included data from the same study populations. In two articles [22,35] participants were recruited from the same survey (n = 799). The study population in Bauman et al. [18] was from the 2009 Arizona Youth Risk Behaviour Survey (Arizona YRBS) (n = 1,491), with Romero et al. [36] focussing on a subsample of Hispanic females (n = 650). Two articles [37,38] included patients from the same hospital. Cénat et al. [39] and Hébert et al. [40] both examined a sample (n = 8,194) surveyed using the 2011 Quebec Youths' Romantic Relationships Survey (QYRRS). The latter included an additional wave of respondents from 2012 (total n = 14,974). Hay and Meldrum [16] and Hay et al. [41] were based on the same population (n = 426). Finally, a trio of papers were based upon the 2011 YRBS: Messias et al. [42] and Reed et al. [43] both taking their sample (n = 15,425) from the national version of the survey, and Kindrick et al. [44] (n = 1,375) examining only respondents from Arkansas.

Specific details on measures included in meta-analysis

Cybervictimisation and self-harm

Eleven independent studies [11,16-18,20,42,48-50,54,55] (n = 85,967) were eligible for meta-analysis of the association between cybervictimisation and SH. Seven articles were rated high quality and four were rated medium. One study [36] reported nonsignificant results on suicide attempt and cybervictimisation, but was excluded as it was a subpopulation of another report [18], as were three others [41,43,44]. Schenk et al. [22] combined suicide attempt and suicide planning, the latter of which would not be considered self-harm, and Schenk et al. [35] combined victims and perpetrators. These combinations could not be disaggregated so both studies were excluded from the meta-analysis. Roh et al. [53] was excluded from meta-analysis since the cybervictimisation exposure was combined with other forms of bullying. Three studies reported more than one outcome eligible for this meta-analysis [17,49,55]. Elgar et al. [49] categorised their results by frequency of cybervictimisation ("rarely", "sometimes", and "often") for SH and for suicide attempts. Results from the "sometimes" category were included as the average exposure in all meta-analyses. Schneider et al. [17] reported an OR = 3·56 [2.95, 4.29] for "self-injury" plus an OR = 5·00 [3·73, 6·71] for "suicide attempt", while Sinclair et al. [55], reported "cutting/self-harm", OR = 2·95, and "suicide attempt", OR = 3·82. The multiple effect sizes for these latter two studies were combined and corresponding variances computed. This technique was also applied to effect sizes presented by gender in Bauman et al. [18]. The meta-analysis produced an OR = 2·35 [1·65, 3·34] (P < ·001), with "high" heterogeneity between studies (P < ·001).

Eleven independent studies [11,16-18,20,42,48-50,54,55] (n = 85,967) were eligible for meta-analysis of the association between cybervictimisation and SH (Manuscript Figure 2, Manuscript Table 1). Seven articles were rated high quality and four were rated medium. The meta-analysis produced OR = 2·35 [1·65, 3·34].

Cybervictimisation and suicidal behaviours

An empirical association between cybervictimisation and suicidal behaviours was identified in 32 articles. Eleven were of high quality, 16 medium, and five low. Regression coefficients ranged from β = 0·15 (P < 0·01) for suicide risk [50] to β = 0·97 (P < 0·001) for suicidal behaviour [51]. Odds ratios ranged from 1·73 [1·26, 2·38] for suicide attempt [54] to 6·32 [1·44, 8·69] for suicidal ideation [47]. Schenk et al. [22] (medium quality) applied a χ^2 goodness-of-fit producing $\chi^2(2, 138)$ = 9·09 (P = 0·03) when the frequency of suicidal planning and attempts

between cybervictims and controls were compared. Three papers found no significant association between cybervictimisation and measures of suicidal behaviours [19,21,36,52,56].

Twenty-one studies [11,16-18,20,22,34,38,39,42,45-52,54,55,57], with 116,616 participants, were included in the meta-analysis (Manuscript Figure 2, Manuscript Table 1). Nine were rated high quality, 11 medium, and one low [38]. A number of studies were excluded from meta-analysis as a subsample of another study [19,21,35-37,40,41,43,44,53,56]. Cénat et al. [39] was included over Hébert et al. [40] since the latter combined suicidal behaviours with two other measures (self-esteem and psychological distress). Turner et al. [56] was excluded since the data presented were unsuitable for calculating the variation, as was Roh et al. [53] for the reasons outlined previously. Combination of effect sizes was again applied for those studies which presented more than one eligible outcome [11,17,42,46,49,54,55] and for Bauman et al. [18], whose results were presented by sex. In Schenk et al. [22], it was necessary to use the available raw data to manually calculate an effect size. The meta-analysis produced an OR = 2·10 [1·73, 2·55] ($P < \cdot001$), with "high" heterogeneity between studies ($P < \cdot001$).

Cybervictimisation and suicide attempt

Ten studies [11,17,18,20,42,48-50,54,55] with 85,541 participants were eligible for inclusion in meta-analysis for this association (Manuscript Figure 2, Manuscript Table 1). The results of Bauman et al. [18] were combined as in the previous meta-analyses. Seven studies were rated high quality and three as medium quality. The summary effect size of the association between cybervictimisation and suicide attempt was OR = 2·57 [1·69, 3·90] ($P < \cdot001$), with "high" heterogeneity between studies ($P < \cdot001$).

Cybervictimisation and suicidal ideation

Sixteen studies [11,16,17,22,34,36,38,39,42,46,47,49,52,54,55,57] with 103,774 participants were included in the meta-analysis for this association (Manuscript Figure 2, Manuscript Table 1). Seven studies were rated high quality, seven medium quality, and two low quality. The summary effect size for this meta-analysis was an OR = 2·15 [1·70, 2·71] ($P < \cdot001$), with "high" heterogeneity between studies ($P < \cdot001$).

Cyberbullying perpetration and suicidal behaviours

The association between cyberbullying perpetration and suicidal behaviours was examined in six papers [11,18,35,36,45,57] (five independent studies [11,35,36,45,57] with 4,062 participants). Bauman et al. [18] did not report a P-value for the direct effect of cyberbullying perpetration on suicide attempt and the confidence interval of its indirect effects were unreliable so a variance could not be calculated. Therefore, Romero et al. [36] was included instead. Of the five studies included in the meta-analysis (Manuscript Figure 3, Manuscript Table 1): one was rated high quality, three medium, and one low. Combination of effect sizes was again applied where appropriate [11]. The summary effect size of this association was an OR = 1·21 [1·02, 1·44] ($P = \cdot03$), with "moderate" heterogeneity between studies ($P = \cdot006$).

Cyberbullying perpetration and suicide attempt

Three articles [11,18,36] examined this association. One [11] found an OR of 1.49 ($P < 0.05$). Bauman et al. [18] reported a direct effect of $\beta = 0\cdot14$ ($P < 0\cdot05$) for males only while a study based on a subsample of its population [36], found no significant effect. Meta-analysis was not conducted for the association between cyberbullying perpetration and suicide attempt since only two studies would be included.

Cyberbullying perpetration and suicidal ideation

Four studies [11,35,36,57], with 3,811 participants, were included in this meta-analysis (Manuscript Figure 3, Manuscript Table 1). Of these one was rated high quality, two medium, and one low. A summary effect size of OR = 1·23 [1·10, 1·37] (P < ·001), was produced for this association. I^2 did not reach the threshold required for "low" heterogeneity (P > = ·27).

All calculated values of I^2 are displayed in Manuscript Table 1.

Meta-regression: Traditional victimisation

Twelve articles [11,17,34,38,39,42,46-48,51,54,57] reported a traditional victimisation prevalence rate and effect size for cybervictimisation and suicidal behaviours, with meta-regression returning unstandardised and standardised coefficients of B = –0·03 and β = –0·84, respectively, with P = ·001. Furthermore, ten [11,17,34,38,39,42,46,47,54,57] articles reported prevalence rates for traditional victimisation as well as the association between cybervictimisation and suicidal ideation, with meta-regression giving an unstandardised coefficient of B = –0·03 and standardised coefficient β = –0·89, with P = ·001.

The results of both meta-regressions are shown in Table 1 of this document. Plots for each are shown in Figure 1 of this document.

Table 1: Results of the two meta-regressions for articles which reported traditional victimisation prevalence rates. *B* is the unstandardised coefficient of the predictor variables; SE is the standard error; *β* is the standardised coefficient of covariate *X*; and *t* is Student's *t*-statistic.

Measure	Term	*B*	SE	*β*	*t*	*P*
**Suicidal behaviours**	Intercept	1·67	0·16	-	10·26	< ·001
	X	–0·03	0·01	–0·84	–4·99	< ·001
**Suicidal ideation**	Intercept	1.75	0·18	-	9.98	< ·001
	X	–0·03	0·01	–0·89	–5·38	< ·001

Figure 1: Plots for the two meta-regressions of traditional victimisation prevalence against study effect size. The red line represents the regression slope. The blue circles represent included studies, with each circle's area proportional to its weight in the regression. The black dashed line represents the equation lnOR = *X*, where *X* is the absolute value of traditional victimisation prevalence. The regression equation is given in each plot.

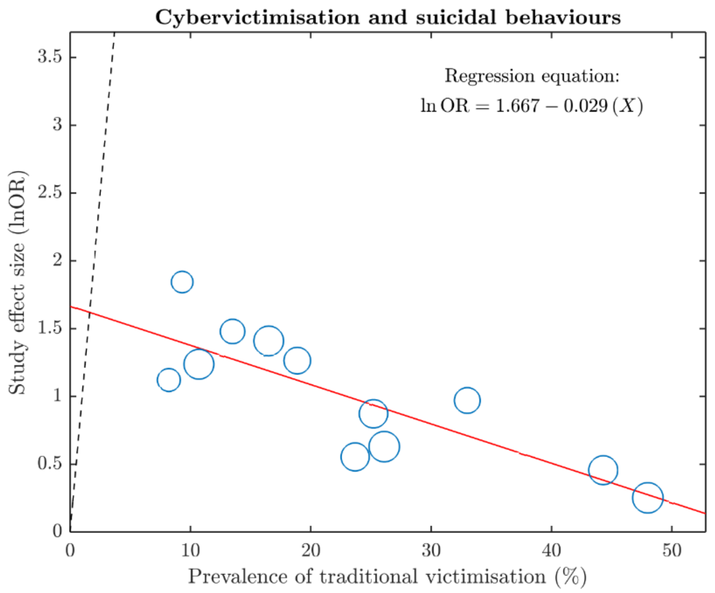

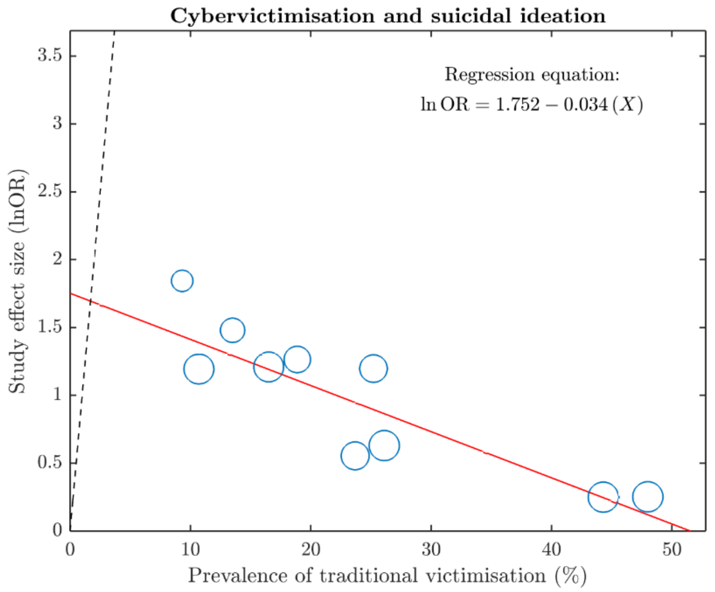

Sensitivity analyses

When sensitivity analysis was performed for school-based settings, little to no change was observed compared to that of the original meta-analyses. The largest change in effect size was for cybervictimisation and suicidal behaviours, whose odds ratio increased from OR = 2·10 [1·73, 2·55] to OR = 2·23 [1·74, 2·86] after six articles that did not use school-based samples were removed, with heterogeneity increasing from I^2 = 92·22% to I^2 = 92·83%. Overall, I^2 did not change by more than 1·78% for the statistically significant analyses. The unchanged high levels of heterogeneity indicate that a large amount of unexplained variance remains across studies, which is not attributable to sample setting in this instance. Full results are shown in Table 2 of this document.

The second sensitivity analysis was based on articles which considered cybervictimisation separately to traditional victimisation or controlled for traditional victimisation. This produced statistically significant results for all four outcomes considered: self-harm, OR = 3·09 [2·36, 4·04]; suicidal behaviours, OR = 2·35 [1·56, 3·54]; suicide attempt, OR = 3·24 [2·37, 4·43]; and suicidal ideation, OR = 2·43 [1·59, 3·71]. Each of these results were higher than their equivalent in the original meta-analyses. Heterogeneity between studies was significantly lower for the outcomes of SH (I^2 = 73·07% compared to I^2 = 94·28% for the original meta-analysis) and suicidal ideation (I^2 = 77·63% compared to I^2 = 94·75% for the original meta-analysis), but did not change by more than 3·61% for suicidal behaviours or suicidal ideation. Full results are shown in Table 2 of this document.

Table 2: Sensitivity analyses (school-based settings only; cybervictimisation vs. traditional victimisation)

Cyberbullying group	Measure	Group analysed	*k*	*n*	OR [95% CI]	*z*	T^2	*Q*	I^2
**Victimisation**	**Self-harm**	Overall	11	85,967	2·35 [1·65, 3·34]	4·76	0·30	174·92	94·28%
		School-based	10	80,538	2·39 [1·62, 3·52]	4·40	0·33	172·48	94·78%
		CV only or control for TV	5	61,045	3·09 [2·36, 4·04]	8·25	0·06	14·86 [a]	73·07%
**Victimisation**	**Suicidal behaviours**	Overall	21	116,616	2·10 [1·73, 2·55]	7·45	0·15	256·94	92·22%
		School-based	15	107,568	2·23 [1·74, 2·86]	6·36	0·19	195·38	92·83%
		CV only or control for TV	8	64,829	2·35 [1·56, 3·54]	4·09	0·29	165·90	95·78%
**Victimisation**	**Suicide attempt**	Overall	10	85,541	2·57 [1·69, 3·90]	4·41	0·39	171·48	94·75%
		School-based	9	80,112	2·64 [1·65, 4·24]	4·02	0·45	168·85	95·26%
		CV only or control for TV	5	61,045	3·24 [2·37, 4·43]	7·35	0·09	17·88 [b]	77·63%
**Victimisation**	**Suicidal ideation**	Overall	16	103,774	2·15 [1·70, 2·71]	6·39	0·17	157·62	90·48%
		School-based	12	100,406	2·06 [1·60, 2·64]	5·64	0·16	142·12	92·26%
		CV only or control for TV	6	58,449	2·43 [1·59, 3·71]	4·11	0·23	84·56	94·09%

All values of *z* and *Q* are significant to the *P* < ·001 level unless otherwise stated; [a] *P* = ·005; [b] *P* = ·001; CV = Cybervictimisation; TV = Traditional victimisation.

Publication bias

The cybervictimisation meta-analysis with the most number of articles (k = 21), was that for suicidal behaviours. The funnel plot for this meta-analysis revealed little to no sign of publication bias: Studies were evenly distributed around the mean at the top of the plot, with only one study in the bottom-right of the plot (with larger than average OR) unmatched to the left. For cyberbullying perpetration, the suicidal behaviours meta-analysis was also used. This showed no signs of publication bias, however, it should be noted that this conclusion is based upon a low number of studies (k = 5). The funnel plots for each meta-analysis are shown in Figure 2 of this document.

Figure 2: Funnel plots for the cybervictimisation and perpetration meta-analyses with the most number of studies The *x*-axis is in units of OR, while the *y*-axis is in units of the standard error (SE_{lnOR}). Included studies are represented by the blue circles; the summary effect size is given by the red dotted line; and the 95% confidence interval for any given value of the standard error is represented by the two diagonal black lines.

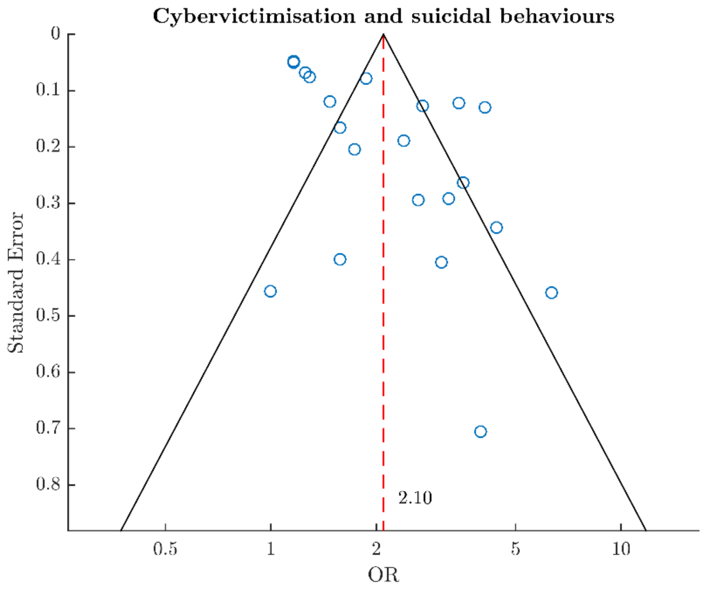

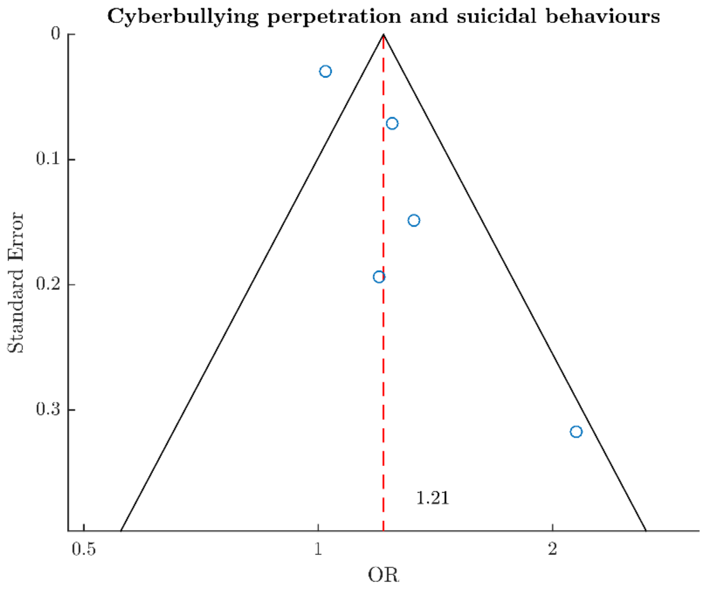

Supplement: Multimedia Appendix 4 [file jmir_v20i4e129_app4.pdf]
