# Supplementary material for: Self-Harm, Suicidal Behaviours, and Cyberbullying in Children and Young People: Systematic Review
Source: J Med Internet Res. 2018 Apr 19;20(4):e129. doi: 10.2196/jmir.9044 (PMC5934539; doi:10.2196/jmir.9044)
Supplement: Multimedia Appendix 5 [file jmir_v20i4e129_app5.pdf]

Multimedia Appendix 5: Summary of Outcome Measures Used in Studies Included in Review

Author<br>Year	Data collection method<br>Outcome measures used
**Questions used for outcome measures**	
Alavi [37]<br>2015	Retrospective review of patient medical records<br>CV, TV, SB, SI
CV – Question(s) to determine cybervictimisation status not specified<br>TV – Question(s) to determine traditional victimisation (split into verbal and physical victimisation) status not specified<br>SB, SI – Question(s) to determine suicidal ideation status not specified<br>CP, TP, SH, SA, MH – None<br>Other measures – History of sexual, physical, and emotional abuse was measured	
Arat [46]<br>2015	Self-report questionnaire<br>CV, TV, SB, SI, MH
CV – One question from the YRBS asking "During the past 12 months, have you ever been electronically bullied?"<br>TV – One question from the YRBS asking "During the past 12 months, have you ever been bullied on school property?"<br>SB, SI – One question from the YRBS asking "During the past 12 months, did you ever seriously consider attempting suicide?"<br>MH – One question from the YRBS asking "During the past 12 months, did you ever feel so sad or hopeless almost every day for two weeks or more in a row that you stopped doing some usual activities?"<br>CP, TP, SH, SA – None<br>Other measures – Several other protective and risk factors were considered such as dietary patterns, TV consumption, and physical activity	
Bannink [34]<br>2014	Self-report questionnaire<br>CV, TV, SB, SI, MH
CV – One question from the RYM asking whether individuals had been bullied in the past four weeks via the Internet or their phone; answers were dichotomised into "Never being victimised" and "Being victimised once or twice<br>TV – One question from the Rotterdam Youth Monitor (RYM) asking whether individuals had been bullied in the past four weeks at school; answers were dichotomised into "Never being victimised" and "Being victimised once or twice<br>SB, SI – One question from the RYM asking "In the past 12 months have you ever seriously considered ending your life?"<br>Other measures – Incorporated as potential confounders were age, gender, ethnicity, and level of education<br>MH – SDQ, 25 items, Dutch self-report version<br>TP, CP, SH, SA – None<br>Other measures – None	
Bauman [18]<br>2013	Self-report questionnaire<br>CV, CP, TV, TP, SH, SB, SA
CV – One question from the Arizona YRBS asking "During the past 12 months, how frequently have you been electronically bullied, such as through e-mail, chat rooms, instant messaging, web sites or text messaging?"<br>CP – One question from the Arizona YRBS asking "During the past 12 months, how frequently have you electronically bullied someone else, such as through e-mail, chat rooms instant messaging, web sites or text messaging?"<br>TV – One question from the Arizona YRBS asking "During the past 12 months, how frequently have you been harassed or bullied on school property?"<br>TP – One question from the Arizona YRBS asking "During the past 12 months, how frequently have you harassed or bullied someone else on school property?"<br>SH – One question from the Arizona YRBS on suicide attempt<br>SB, SA – One question from the Arizona YRBS<br>Other measures – Gender, grade level and ethnicity were also analysed to assess whether there was an association with depression or suicidality<br>MH – One question from the Arizona YRBS asking "During the past 12 months, did you ever feel so sad or hopeless almost every day for two weeks or more in a row that you stopped doing some usual activities?"<br>SI – None<br>Other measures – None	
Bonanno [57]<br>2013	Self-report questionnaire<br>CV, CP, TV, TP, SB, SI, MH

<table>
<tr><td colspan="2">CV, CP, TV, TP – Assessed via a 10-item self-report adapted from measures originally developed by Olweus (1993) in which involvement in traditional bullying is split into the three subcategories: physical, verbal, and social
SB, SI – SIQ-JR, 15 items
MH – Depressive symptomatology examined using CES-D scale, 20 items
SH, SA – None
Other measures – None</td></tr>
<tr><td>Cassidy [19]
2009</td><td>Self-report questionnaire
CV, CP, SB, SI</td></tr>
<tr><td colspan="2">CV – Eighteen different questions relating to different examples of cybervictimisation
CP – Participants were asked if they have “harassed or bullied another student online either through chat rooms, emails, text messaging or any other online discussions”.
SB, SI – Identified from responses to open-ended questions
TV, TP, SH, SA, MH – None</td></tr>
<tr><td>Cénat [39]
2015</td><td>Self-report questionnaire
CV, TV, SB, SI</td></tr>
<tr><td colspan="2">CV – One question from the QYRRS asking “How many times has someone bullied you (rumors, intimidation, threatening, etc.) using the Internet (Facebook, MySpace, MSN, email, texto, etc.)?”
TV – One question from the QYRRS asking “How many times has someone bullied you at school or elsewhere except via the Internet?”
SB, SI – One question from the QYRRS asking “Have you ever seriously thought of committing suicide?”
CP, TP, SH, SA, MH – None
Other measures – Psychological distress, self-esteem, and homophobic bullying were also measured</td></tr>
<tr><td>DeSmet [47]
2014</td><td>Self-report questionnaire
CV, TV, SB, SI</td></tr>
<tr><td colspan="2">CV – Participants were asked on the HBSC if they had been a victim in cyberbullying (“via Internet, e.g. chat or e-mail, or via mobile phone, e.g. text messages”) with answer options on a 5-point Likert scale
TV – Participants were asked on the HBSC if they been a victim in traditional bullying (“bullied at school”) with answer options on a 5-point Likert scale
SB, SI – One question from the HBSC asking “Have you ever thought about ending your life?” with answer options on a 5-point Likert scale
CP, TP, SH, SA, MH – None
Other measures – Obesity, family affluence, quality of life, self-esteem, and barriers and facilitators to a healthy diet were also measured</td></tr>
<tr><td>Duong [48]
2014</td><td>Self-report questionnaire
CV, TV, SH, SB, SA</td></tr>
<tr><td colspan="2">CV – One question from the YRBS asking “During the past 12 months, have you ever been electronically bullied, such as through e-mail, chat rooms, instant messaging, Web sites, or text messaging?”
TV – One question from the YRBS asking “During the past 12 months, have you ever been bullied on school property?”
SH, SA – One question from the YRBS asking “During the past 12 months, how many times did you actually attempt suicide?” with answer options “0 times”, “1 time”, “2 or 3 times”, “4 or 5 times”, or “6 or more times”
SB, SI, MH – None
Other measures – Aggressive behaviour, sexual orientation, and connection with a school teacher were also measured</td></tr>
<tr><td>Elgar [49]
2014</td><td>Self-report questionnaire
CV, TV, TP, SH, SB, SA, SI</td></tr>
<tr><td colspan="2">CV – One question from the DCYA asking “In the past 12 months, how often have you been bullied, threatened, or harassed through the Internet or text messaging?" with answer choices "Never, rarely, sometimes, or often"
TV – Four-item victimization subscale of The Bullying and Victimisation subscales of the University of Illinois Aggression Scales
TP – Six-item bullying subscale of The Bullying and Victimisation subscales of the University of Illinois Aggression Scales
SH, SA – One question from the DCYA asking youth if they had intentionally hurt themselves, such as cutting or burning, in the past 12 months; and one question from DCYA regarding suicide attempt
SB, SA, SI – Two questions from DCYA regarding suicidal thoughts and suicide attempts
MH – Two questions from the DCYA asking youth whether they have had feelings of anxiety or depression
CP – None
Other measures – Family dinners, externalising problems, and substance use problems</td></tr>
<tr><td>Fu [33]
2014</td><td>Researcher-completed questionnaire
CV, TV</td></tr>
<tr><td colspan="2">CV – One question from the EUKOS with respondent confirming that they have had “the experience of online bullying” but answered</td></tr>
</table>

<table>
<tr><td colspan="2">“no” when asked if the experience had occurred “in person, face-to-face” and “by mobile phone calls, texts, or image/video texts”
TV – Assessed as part of EUKOS but no measure given for exclusive traditional bullying victimisation
TP, CP, SH, SB, SA, SI, MH – None
Other measures – Unnatural child deaths, exposure to self-harm/suicide information, and Internet addiction</td></tr>
<tr><td>Goebert [20]
2011</td><td>Self-report questionnaire
CV, SH, SB, SA, MH</td></tr>
<tr><td colspan="2">CV – Participants were asked by which method they had experienced cybervictimisation: (1) text; (2) e-mail; (3) web; (4) cybercontrol of victim’s phone by victim’s partner; (5) cybercontrol of victim’s personal website by victim’s partner
SH, SA – Participants were asked “how many suicide attempts they had made in the past 12 months”
MH – Depression was measured using two questions from the CES-D
TV, CP, TP, SB, SI – None
Other measures – Substance abuse and type was also measured (1) binge drinking; (2) marijuana</td></tr>
<tr><td>Hay [16]
2010a</td><td>Self-report questionnaire
CV, TV, SH, SB, SI</td></tr>
<tr><td colspan="2">CV – Three-item scale asking how often respondents had been the victim of different kinds of cyberbullying during the past 12 months, with answers categorised as “never”, “rarely”, “sometimes”, or “often”
TV – Six-item scale asking how often respondents had been the victim of different kinds of traditional bullying during the past 12 months, with answers categorised as “never”, “rarely, “sometimes”, or “often”
SH – One question asking how often “you purposely hurt yourself without wanting to die” such as “cutting or burning” with answers categorised as “never”, “rarely, “sometimes”, or “often”
SB, SI – One question asking how often “you think about killing yourself” with answers categorised as “never”, “rarely, “sometimes”, or “often”
CP, TP, SA, MH – None
Other measures – None</td></tr>
<tr><td>Hay [41]
2010b</td><td>Self-report questionnaire
CV, TV, SH, SB, SI</td></tr>
<tr><td colspan="2">CV – Three-item scale asking how often respondents had been the victim of different kinds of cyberbullying during the past 12 months, with answers categorised as “never”, “rarely”, “sometimes”, or “often”
TV – Six-item scale asking how often respondents had been the victim of different kinds of traditional bullying during the past 12 months, with answers categorised as “never”, “rarely, “sometimes”, or “often”
SH – One question asking how often “you purposely hurt yourself without wanting to die” such as “cutting or burning” with answers categorised as “never”, “rarely, “sometimes”, or “often”
SB, SI – One question asking how often “you think about killing yourself” with answers categorised as “never”, “rarely, “sometimes”, or “often”
CP, TP, SA, MH – None
Other measures – Externalising delinquency was also measured</td></tr>
<tr><td>Hébert [40]
2016</td><td>Self-report questionnaire
CV, TV, SB, SI</td></tr>
<tr><td colspan="2">CV – One question from the QYRRS asking “How many times did someone harass you (with rumors, intimidation, threats, etc.) electronically (Facebook, MySpace, MSN, emails, texts, etc.)”
TV – One question from the QYRRS asking “How many times did someone harass you (with rumours, intimidation, threats, etc.) at school or elsewhere (excluding electronically)?”
SB, SI – One question asking “In the past 12 months, have you ever seriously thought of committing suicide?”
CP, TP, SH, SA, MH – None
Other measures – Psychological distress, maternal support, and lifetime child sexual abuse were also measured</td></tr>
<tr><td>Hinduja [11]
2010</td><td>Self-report questionnaire
CV, CP, TV, TP, SH, SB, SA, SB, SI</td></tr>
<tr><td colspan="2">CV – Nine-item scale asking students about their experiences of various forms of cybervictimisation in the last 30 days, with answers categorised in terms of frequency
CP – Five-item scale asking students about their experiences of various forms of cyberbullying perpetration in the last 30 days, with answers categorised in terms of frequency
TV – Ten-item scale asking students about their experiences of various forms of traditional victimisation in the last 30 days, with answers categorised in terms of frequency
TP – Ten-item scale asking students about their experiences of various forms of traditional perpetration in the last 30 days, with answers categorised in terms of frequency
SH, SB, SA – One of four items adapted from the NASHS asking students if they have ever attempted suicide with answers dichotomised to “yes” or “no”
SB, SI – Four-item scale adapted from the NASHS
MH – None</td></tr>
</table>

Other measures – None	
Kindrick [44]<br>2013	Self-report questionnaire<br>CV, TV, SH, SB, SA, SARMT, SI, SP MH
CV – Three questions from the Arkansas YRBS<br>TV – Three questions from the Arkansas YRBS<br>SH – One question from Arkansas YRBS<br>SB, SA, SARMT, SI, SP – Four questions from the Arkansas YRBS<br>MH – One question from the Arkansas YRBS asking “During the past 12 months, did you ever feel so sad or hopeless almost every day for two weeks or more in a row that you stopped doing some usual activities?”<br>TP, CP - None<br>Other measures – None	
Kodish [50]<br>2016	Self-report questionnaire<br>CV, TV, SB, SH, SA, MH
CV – One question from the BHS asking “How often are you cyber bullied – chat rooms, Facebook, instant messaging, text messages on your cell phone?”<br>TV – Two questions from the BHS assessing physical and verbal victimisation<br>SB – Four items measuring suicide risk<br>SH, SA – One question asking whether the participant had ever attempted suicide<br>MH – Depression was measured using a 5-item scale<br>CP, TP – None<br>Other measures – None	
Litwiller [51]<br>2013	Self-report questionnaire<br>CV, TV, SB
CV – Three questions devised by a “local coalition”<br>TV – Three questions from the YRBS<br>SB– Four questions from the YRBS including questions of both SA and SI, but presented as the single outcome “suicidal behaviour”<br>TP, CP, SH, SA, SI, MH - None<br>Other measures – Physical bullying, substance use, violent behaviour, and sexual behaviour	
Messias [42]<br>2014	Self-report questionnaire<br>CV, TV, SH, SB, SA, SARMT, SI, SP
CV – One question from the YRBS asking “During the past 12 months, have you ever been electronically bullied? (Include being bullied through e-mail, chat rooms, instant messaging, Web sites, or texting)”<br>TV – One question from the YRBS asking “During the past 12 months, have you ever been bullied on school property?”<br>SH – One question from the YRBS<br>SB, SA, SARMT, SI, SP – Four questions from the YRBS<br>MH – One question from the YRBS asking “During the past 12 months, did you ever feel so sad or hopeless almost every day for two weeks or more in a row that you stopped doing some usual activities?”<br>TP, CP – None<br>Other measures – None	
Mitchell [52]<br>2014	Researcher-completed questionnaire<br>CV, SB, SI, MH
CV – Questions from YISS-3 in which youth were asked whether they had been victims of various kinds of harassment or abuse, including online harassment<br>SB, SI – One question from the TSCC asking “In the last month, how often have you been wanting to kill yourself?”<br>MH – Depression subscale of the TSCC, 9 items<br>TV, TP, CP, SH, SA – None<br>Other measures – Forms of victimisation other than online harassment, problem behaviour/conflict, and various demographic characteristics (age, gender, etc.)	
Price [21]<br>2010	Self-report questionnaire<br>CV, CP, SH, SB, SI
CV – Participants were asked if they had been cybervictimised, with this defined as being “teased, harassed, ignored or bullied in some other way via an electronic device (e.g. mobile phone or computer)”<br>CP – Question used to determine cyberbullying perpetration status unspecified<br>SH – Identified from responses to open-ended questions	

<table>
<tr><td colspan="2">SB, SI – Identified from responses to open-ended questions
TV, TP, SA, MH – None</td></tr>
<tr><td>Reed [43]
2015</td><td>Self-report questionnaire
CV, TV, SH, SB, SA, SI, SP</td></tr>
<tr><td colspan="2">CV – One question from the YRBS asking “During the past 12 months, have you ever been electronically bullied? (Include being bullied through e-mail, chat rooms, instant messaging, Web sites, or texting)”
TV – One question from the YRBS asking “During the past 12 months, have you ever been bullied on school property?”
SH, SA – One question from the YRBS asking “During the past 12 months, how many times did you actually attempt suicide?”
SB, SA, SI, SP – Three questions from the YRBS
MH – One question from the YRBS asking “During the past 12 months, did you ever feel so sad or hopeless almost every day for two weeks or more in a row that you stopped doing some usual activities?”
TP, CP – None
Other measures – None</td></tr>
<tr><td>Roberts [38]
2016</td><td>Retrospective review of patient medical records
CV, TV, SB, SI</td></tr>
<tr><td colspan="2">CV – Question(s) to determine cybervictimisation status not specified
TV – Question(s) to determine traditional victimisation (split into verbal and physical victimisation) status not specified
SB, SI – Question(s) to determine suicidal ideation status not specified
CP, TP, SH, SA, MH – None
Other measures – Substance abuse and history of sexual, physical, and emotional abuse were also measured</td></tr>
<tr><td>Roh [53]
2015</td><td>Self-report questionnaire
CV, TV, SB, SI, SH, SA</td></tr>
<tr><td colspan="2">CV – Participants were asked if they had been the victim of cyberbullying during the past 12 months
TV – Participants were asked if they had been the victim of physical or verbal bullying during the past 12 months
SB, SI – One question asking “I have said that I wanted to die during the past 3 months”
SH, SA – One question asking “I have attempted suicide during the past 3 months”
CP, TP, MH – None
Other measures – Ostracism, harassment, extortion, and sexual bullying were also measured</td></tr>
<tr><td>Romero [36]
2013</td><td>Self-report questionnaire
CV, CP, TV, TP, SH, SB, SA, SI, SP, MH</td></tr>
<tr><td colspan="2">CV – One question from the Arizona YRBS asking “During the past 12 months, how frequently have you been electronically bullied, such as through email, chat rooms, instant messaging, web sites, or text messaging?”
CP – One question from the Arizona YRBS asking “During the past 12 months, how frequently have you electronically bullied someone else, such as through email, chat rooms, instant messaging, web sites, or text messaging?”
TV – One question from the Arizona YRBS asking “During the past 12 months, how frequently have you been harassed or bullied on school property?”
TP – One question from the Arizona YRBS asking “During the past 12 months, how frequently have you harassed or bullied someone else on school property?”
SH – One question from the Arizona YRBS
SB, SA, SI, SP – Three questions from the Arizona YRBS
MH – One question from the Arizona YRBS asking “During the past 12 months, did you ever feel so sad or hopeless almost every day for two weeks or more in a row that you stopped doing some usual activities?” Other measures – None</td></tr>
<tr><td>Sampasa-Kanyinga [54]
2014</td><td>Self-report questionnaire
CV, TV, SB, SI, SH, SA, SP, MH</td></tr>
<tr><td colspan="2">CV – One question from the Eastern Ontario YRBS asking “During the past 12 months, have you ever experienced cyberbullying, that is, being bullied by email, text messaging, instant messaging, social networking or another website?”
TV – One question from the Eastern Ontario YRBS asking “During the past 12 months, have you ever been bullied or threatened by someone while on school property?”
SB, SI – One question from the Eastern Ontario YRBS asking “During the past 12 months, did you ever seriously consider attempting suicide?”
SH, SA – One question from the Eastern Ontario YRBS asking “If you attempted suicide during the past 12 months, did any attempt result in injury, poisoning, or overdose that had to be treated by a doctor or nurse?”. Responses were analysed as having attempted suicide or not having attempted suicide, regardless of medical treatment
SP – One question from the Eastern Ontario YRBS asking “During the past 12 months, did you make a plan about how you would attempt suicide?”
MH – One question from the Eastern Ontario YRBS asking “During the past 12 months, did you ever feel so sad or hopeless almost every day for 2 weeks or more in a row that you stopped doing some usual activities?”
CP, TP – None</td></tr>
</table>

Other measures – Computer use, sedentary activities, and substance abuse were also measured	
Schenk [22]<br>2012	Self-report questionnaire<br>CV, CP, SH, SB, SA, SI, SP, MH
CV – Various questions regarding cybervictimisation from a 47-item questionnaire called the Internet Experiences Questionnaire (IEQ) developed by the researchers for the purpose of the study, comprising questions on both cybervictimisation and cyberbullying perpetration. The IEQ was not validated<br>CP – Although measures were taken on cyberbullying perpetration using the IEQ, these were analysed in a separate paper (Schenk et al. [35])<br>SH – One question from the SBQ-R<br>SB, SA, SI, SP – Four questions from the SBQ-R<br>MH – SCL-90-R which assess nine symptoms of psychopathology: somatisation, obsessive compulsiveness, interpersonal sensitivity, depression, anxiety, hostility, phobic anxiety, paranoid ideation, and psychoticism<br>TV, TP, – None<br>Other measures – None	
Schenk [35]<br>2013	Self-report questionnaire<br>CV, CP, SH, SB, SA, SI, SP, MH
CV – Although measures were taken on cybervictimisation using the IEQ, these were analysed in a separate paper (Schenk et al. [22])<br>CP – Various questions regarding cyberbullying perpetration from the IEQ<br>SH – One question from the SBQ-R<br>SB, SA, SI, SP – Four questions from the SBQ-R<br>MH – SCL-90-R<br>TV, TP – None<br>Other measures – None	
Schneider [17]<br>2012	Self-report questionnaire<br>CV, TV, SH, SB, SA, SARMT, SI, MH
CV – One question from the Massachusetts YRBS asking ‘‘How many times has someone used the Internet, a phone, or other electronic communications to bully, tease, or threaten you?’’<br>TV – One question from the Massachusetts YRBS asking ‘‘During the past 12 months, how many times have you been bullied on school property?’’<br>SH, SA – One question from the MetroWest Adolescent Health Survey, based on the Massachusetts YRBS: "How many times did you hurt or injure yourself on purpose? (For example, by cutting, burning, or bruising yourself on purpose); and two question from the Massachusetts YRBS on suicide attempt and suicide attempt requiring medical treatment<br>SB, SA, SARMT, SI – Four questions from the Massachusetts YRBS<br>MH – One question from the Massachusetts YRBS asking “During the past 12 months, did you ever feel so sad or hopeless almost every day for two weeks or more in a row that you stopped doing some usual activities?”<br>TP, CP – None<br>Other measures – Various demographic characteristics	
Sinclair [55]<br>2012	Self-report questionnaire<br>CV, TV, SH, SA, SB, SI, MH
CV – Various questions from the DCYA<br>TV – Various questions from the DCYA<br>SH, SA – One question from the DCYA asking youth if they had intentionally hurt themselves, such as cutting or burning, in the past 12 months; and one question on suicide attempt in the past 12 months<br>SB, SA, SI – Items assessing suicidal ideation in the past 30 days and suicide attempt in the past 12 months<br>MH – Various questions from the DCYA covering mental health problems such as depression and panic symptoms<br>TP, CP – None<br>Other measures – Demographic characteristics (additional questions to assess sexual orientation), substance use, and school-related problems	
Turner [56]<br>2013	Self-report questionnaire<br>CV, TV, SB, SI, MH
CV, TV – A needs assessment was conducted by Iredell-Statesville School System, while in consultation with the research team, which used two items to assess three different types of bullying: physical, verbal, and cyber-<br>SB, SI – Two questions as part of needs assessment<br>MH – Depression measured by a shortened version of the CES-D, 5 items<br>TP, CP, SH, SA – None<br>Other measures – Demographic characteristics, grades, neighbourhood disorder, parental investment, school involvement, alcohol frequency, marijuana frequency, and other drug frequency	

Yen [45]<br>2014	Self-report questionnaire<br>CV, CP, TV, TP, SB, SI, MH
CV – Three questions from the CEQ<br>CP – Three questions from the CEQ<br>TV – Four questions regarding passive and active traditional victimisation from the C-SBEQ<br>TP – Four questions regarding passive and active traditional perpetration from the C-SBEQ<br>SB, SI – K-SADS-E, 5 items<br>MH – Depression was assessed using the Mandarin version of the CES-D, 20 items. Anxiety was also measured using the MASC-T, 39 items<br>SH, SA – None<br>Other measures – Demographic characteristics, ADHD factors, psychiatric comorbidity, family factors, and peer factors	
**Abbreviations**: CV = Cybervictimisation; CP = Cyberbullying perpetration; TV = Traditional victimisation; TP = Traditional perpetration; SH = Self-harm; SB = Suicidal behaviours; SA = Suicide attempt; SARMT = Suicide attempt requiring medical treatment; SI = Suicidal ideation; SP = Suicide plan; MH = Mental health problems; BHS = Behavioural Health Screen; C-SBEQ = School Bullying Experience Questionnaire (Chinese version); CES-D = Centre for Epidemiologic Studies Depression Scale; CEQ = Cyberbullying Experiences Questionnaire; DCYA = Dane County Youth Assessment; EUKOS = EU Kids Online Survey; HBSC = Health Behaviour in School-aged Children; K-SADS-E = Kiddie-Schedule for Affective Disorders and Schizophrenia (Epidemiological version); MASC-T = Multidimensional Anxiety Scale for Children (Taiwanese version); NASHS = National Adolescent Student Health Survey; QYRRS = Quebec Youths' Romantic Relationships Survey; RYM = Rotterdam Youth Monitor; SBQ-R = Suicide Behaviours Questionnaire-Revised; SCL-90 = Symptom Checklist-90-Revised; SDQ = Strengths and Difficulties Questionnaire; SIQ-JR = Suicidal Ideation Questionnaire Junior; TSCC = Trauma Symptom Checklist for Children; YISS-3 = Third Youth Internet Safety Survey; YRBS = Youth Risk Behaviour Survey	

References

11. Hinduja S, Patchin JW. Bullying, cyberbullying, and suicide. Arch Suicide Res 2010;14(3):206-21. PMID:20658375
16. Hay C, Meldrum R. Bullying victimization and adolescent self-harm: Testing hypotheses from general strain theory. J Youth Adolesc 2010a;39(5):446-59. PMID:20072852
17. Schneider SK, O'Donnell L, Stueve A, Coulter RW. Cyberbullying, school bullying, and psychological distress: A regional census of high school students. Am J Public Health 2012;102(1):171-7. PMID:22095343
18. Bauman S, Toomey RB, Walker JL. Associations among bullying, cyberbullying, and suicide in high school students. J Adolesc 2013;36(2):341-50. PMID:23332116
19. Cassidy W, Jackson M, Brown KN. Sticks and stones can break my bones, but how can pixels hurt me? Students' experiences with cyber-bullying. Sch Psychol Int 2009;30(4):383-402. doi:10.1177/0143034309106948
20. Goebert D, Else I, Matsu C, Chung-Do J, Chang JY. The impact of cyberbullying on substance use and mental health in a multiethnic sample. Matern Child Health J 2011;15(8):1282-6. PMID:20824318
21. Price M, Dalgleish J. Cyberbullying: Experiences, impacts and coping strategies as described by Australian young people. Youth Studies Australia 2010;29(2):51-9.
22. Schenk AM, Fremouw WJ. Prevalence, psychological impact, and coping of cyberbully victims among college students. J Sch Violence 2012;11(1):21-37. doi:10.1080/15388220.2011.630310
33. Fu K-w, Chan C-h, Ip P. Exploring the relationship between cyberbullying and unnatural child death: an ecological study of twenty-four European countries. BMC Pediatr 2014;14(1):195. PMID:25079144
34. Bannink R, Broeren S, van de Looij–Jansen PM, de Waart FG, Raat H. Cyber and traditional bullying victimization as a risk factor for mental health problems and suicidal ideation in adolescents. PLoS One 2014;9(4):e94026. PMID:24718563
35. Schenk AM, Fremouw WJ, Keelan CM. Characteristics of college cyberbullies. Comput Human Behav 2013;29(6):2320-7. doi:10.1016/j.chb.2013.05.013
36. Romero AJ, Wiggs CB, Valencia C, Bauman S. Latina teen suicide and bullying. Hisp J Behav Sci 2013;35(2):159-73. doi:10.1177/0739986312474237
37. Alavi N, Roberts N, Sutton C, Axas N, Repetti L. Bullying victimization (being bullied) among adolescents referred for urgent psychiatric consultation: prevalence and association with suicidality. Can J Psychiatry 2015;60(10):427-31. PMID:26720189
38. Roberts N, Axas N, Nesdole R, Repetti L. Pediatric Emergency Department Visits for Mental Health Crisis: Prevalence of Cyber-Bullying in Suicidal Youth. Child Adolesc Social Work J 2016;33(5):469-72. doi:10.1007/s10560-016-0442-8

39. Cénat JM, Blais M, Hébert M, Lavoie F, Guerrier M. Correlates of bullying in Quebec high school students: The vulnerability of sexual-minority youth. J Affect Disord 2015;183:315-21. PMID:26047959
40. Hébert M, Cénat JM, Blais M, Lavoie F, Guerrier M. Child sexual abuse, bullying, cyberbullying, and mental health problems among high schools students: a moderated mediated model. Depress Anxiety 2016;33(7):623-9. PMID:27037519
41. Hay C, Meldrum R, Mann K. Traditional bullying, cyber bullying, and deviance: A general strain theory approach. J Contemp Crim Justice 2010b;26(2):130-47. doi:10.1177/1043986209359557
42. Messias E, Kindrick K, Castro J. School bullying, cyberbullying, or both: Correlates of teen suicidality in the 2011 CDC youth risk behavior survey. Compr Psychiatry 2014;55(5):1063-8. PMID:24768228
43. Reed KP, Nugent W, Cooper RL. Testing a path model of relationships between gender, age, and bullying victimization and violent behavior, substance abuse, depression, suicidal ideation, and suicide attempts in adolescents. Child Youth Serv Rev 2015;55:128-37. doi:10.1016/j.childyouth.2015.05.016
44. Kindrick K, Castro J, Messias E. Sadness, suicide, and bullying in Arkansas: results from the Youth Risk Behavior Survey--2011. J Ark Med Soc 2013;110(5):90-1. PMID:24383197
45. Yen C-F, Chou W-J, Liu T-L, Ko C-H, Yang P, Hu H-F. Cyberbullying among male adolescents with attention-deficit/hyperactivity disorder: Prevalence, correlates, and association with poor mental health status. Res Dev Disabil 2014;35(12):3543-53. PMID:25241113
46. Arat G. Emerging protective and risk factors of mental health in Asian American students: findings from the 2013 Youth Risk Behavior Survey. Vulnerable Child Youth Stud 2015;10(3):192-205. doi:10.1080/17450128.2015.1045437
47. DeSmet A, Deforche B, Hublet A, Tanghe A, Stremersch E, De Bourdeaudhuij I. Traditional and cyberbullying victimization as correlates of psychosocial distress and barriers to a healthy lifestyle among severely obese adolescents – a matched case–control study on prevalence and results from a cross-sectional study. BMC Public Health 2014;14(1):224. PMID:24593118
48. Duong J, Bradshaw C. Associations between bullying and engaging in aggressive and suicidal behaviors among sexual minority youth: The moderating role of connectedness. J Sch Health 2014;84(10):636-45. PMID:25154527
49. Elgar FJ, Napoletano A, Saul G, Dirks MA, Craig W, Poteat VP, Holt M, Koenig BW. Cyberbullying victimization and mental health in adolescents and the moderating role of family dinners. JAMA Pediatr 2014;168(11):1015-22. PMID:25178884
50. Kodish T, Herres J, Shearer A, Atte T, Fein J, Diamond G. Bullying, depression, and suicide risk in a pediatric primary care sample. Crisis 2016. PMID:27040126
51. Litwiller BJ, Brausch AM. Cyber bullying and physical bullying in adolescent suicide: the role of violent behavior and substance use. J Youth Adolesc 2013;42(5):675-84. PMID:23381779
52. Mitchell KJ, Wells M, Priebe G, Ybarra ML. Exposure to websites that encourage self-harm and suicide: Prevalence rates and association with actual thoughts of self-harm and thoughts of suicide in the United States. J Adolesc 2014;37(8):1335-44. PMID:25313930
53. Roh B-R, Yoon Y, Kwon A, Oh S, Lee SI, Ha K, Shin YM, Song J, Park EJ, Yoo H. The structure of co-occurring bullying experiences and associations with suicidal behaviors in Korean adolescents. PLoS One 2015;10(11):e0143517. PMID:26619356
54. Sampasa-Kanyinga H, Roumeliotis P, Xu H. Associations between cyberbullying and school bullying victimization and suicidal ideation, plans and attempts among Canadian schoolchildren. PLoS One 2014;9(7):e102145. PMID:25076490
55. Sinclair KO, Bauman S, Poteat VP, Koenig B, Russell ST. Cyber and bias-based harassment: Associations with academic, substance use, and mental health problems. J Adolesc Health 2012;50(5):521-3. PMID:22525118
56. Turner MG, Exum ML, Brame R, Holt TJ. Bullying victimization and adolescent mental health: General and typological effects across sex. J Crim Justice 2013;41(1):53-9. doi:10.1016/j.jcrimjus.2012.12.005
57. Bonanno RA, Hymel S. Cyber bullying and internalizing difficulties: Above and beyond the impact of traditional forms of bullying. J Youth Adolesc 2013;42(5):685-97. PMID:23512485
