# Supplementary material for: Self-Harm, Suicidal Behaviours, and Cyberbullying in Children and Young People: Systematic Review
Source: J Med Internet Res. 2018 Apr 19;20(4):e129. doi: 10.2196/jmir.9044 (PMC5934539; doi:10.2196/jmir.9044)
Supplement: Multimedia Appendix 6 [file jmir_v20i4e129_app6.pdf]

Multimedia Appendix 6: Cybervictimization Weighted Prevalence Rates and Prevalence Rates by Study

In some cases, manual calculations have been performed to estimate missing data (these have been marked with explanatory comments).

Study	*n* (total)	*n* (female)	*n* (male)	Total CV % [95 % CI]
**Alavi [37][c,d]**	375	-	-	5·6 [3·7, 8·4]
**Arat [46][d,e]**	10,563	5,135	5,428	14·4 [13·8, 15·1]
**Bannink [34][d]**	8,271	4,003	4,268	6·3 [5·8, 6·9]
**Bauman [18][a,f]**	1,491	725	757	-
**Bonanno [57]**	399	228	171	11·0 [8·3, 14·5]
**Cassidy [19][e]**	365	215	150	-
**Cénat [39][c]**	8,194	-	-	22·9 [22·0, 23·8]
**DeSmet [47][d]**	204	126	78	3·4 [1·7, 6·9]
**Duong [48]**	951	-	-	9·7 [8·0, 11·8]
**Elgar [49][d,f]**	18,834	9,488	9,287	19·3 [18·7, 19·8]
**Fu [33][a]**	25,142	-	-	-
**Goebert [20][d]**	677	416	256	56·1 [52·4, 59·8]
**Hay [16][a,e]**	426	213	213	-
**Hay [41][a,e]**	424	212	212	-
**Hébert [40]**	14,974	-	-	17·5 [16·9, 18·1]
**Hinduja [11][e,g]**	1,963	983	978	29·4 [27·4, 31·5]
**Kindrick [44][a,c]**	1,375	-	-	6·2 [5·0, 7·6]
**Kodish [50]**	5,429	3,067	2,362	5·1 [4·5, 5·7]
**Litwiller [51][e]**	4,693	2,205	2,205	23·0 [21·8, 24·2]
**Messias [42]**	15,425	-	-	6·8 [6·4, 7·2]
**Mitchell [52][d]**	1,560	785	775	11·4 [9·9, 13·0]
**Price [21]**	548	101	447	33·0 [29.2, 37.0]
**Reed [43][a,c,f]**	15,425	7,708	7,656	-
**Roberts [38]**	805	-	-	13·5 [11·3, 16·0]
**Roh [53][e]**	4,410	2,505	1,905	3·0 [2·5, 3·6]
**Romero [36][b,c]**	N/A	650	N/A	N/A
**Sampasa-Kanyinga [54]**	2,999	1,658	1,341	17·4 [16·1, 18·8]
**Schenk [22][d]**	799	572	227	8·6 [6·9, 10·8]
**Schenk [35][b]**	799	572	227	N/A
**Schneider [17][f]**	20,406	10,218	10,050	6·4 [6·1, 6·7]
**Sinclair [55][a,e]**	17,366	8,683	8,683	-
**Turner [56][e]**	2,523	1,287	1,236	13·0 [11·7, 14·4]
**Yen [45][b]**	N/A	N/A	251	N/A
**Weighted prevalence**				12·6 [12·4, 12·7]

CV% = Cybervictimised percentage; [a] Not included in Total Weighted CV% calculation because a total population CV% is not given, [b] Not included in Total Weighted CV% calculation because demographic is not applicable; [c] Not included in Total Weighted CV% calculation because population is a subsample of another study population; [d] Total population CV% calculated manually from available data; [e] Number of females and number of males manually estimated from available data; [f] Sum of number of females and number of males does not equal total population (e.g. due to incomplete survey data or due to participant identifying as neither female nor male); [g] Study categorises CV% in

terms of medium of cybervictimisation (e.g., e-mail and instant messaging). Of the choices available, “One or more of the above, two or more times” was used for the purposes of the weighted prevalence calculation; Dash (-) represents where a study gave no data of the type indicated.

References

11. Hinduja S, Patchin JW. Bullying, cyberbullying, and suicide. Arch Suicide Res 2010;14(3):206-21. PMID:20658375
16. Hay C, Meldrum R. Bullying victimization and adolescent self-harm: Testing hypotheses from general strain theory. J Youth Adolesc 2010a;39(5):446-59. PMID:20072852
17. Schneider SK, O'Donnell L, Stueve A, Coulter RW. Cyberbullying, school bullying, and psychological distress: A regional census of high school students. Am J Public Health 2012;102(1):171-7. PMID:22095343
18. Bauman S, Toomey RB, Walker JL. Associations among bullying, cyberbullying, and suicide in high school students. J Adolesc 2013;36(2):341-50. PMID:23332116
19. Cassidy W, Jackson M, Brown KN. Sticks and stones can break my bones, but how can pixels hurt me? Students’ experiences with cyber-bullying. Sch Psychol Int 2009;30(4):383-402. doi:10.1177/0143034309106948
20. Goebert D, Else I, Matsu C, Chung-Do J, Chang JY. The impact of cyberbullying on substance use and mental health in a multiethnic sample. Matern Child Health J 2011;15(8):1282-6. PMID:20824318
21. Price M, Dalgleish J. Cyberbullying: Experiences, impacts and coping strategies as described by Australian young people. Youth Studies Australia 2010;29(2):51-9.
22. Schenk AM, Fremouw WJ. Prevalence, psychological impact, and coping of cyberbully victims among college students. J Sch Violence 2012;11(1):21-37. doi:10.1080/15388220.2011.630310
33. Fu K-w, Chan C-h, Ip P. Exploring the relationship between cyberbullying and unnatural child death: an ecological study of twenty-four European countries. BMC Pediatr 2014;14(1):195. PMID:25079144
34. Bannink R, Broeren S, van de Looij–Jansen PM, de Waart FG, Raat H. Cyber and traditional bullying victimization as a risk factor for mental health problems and suicidal ideation in adolescents. PLoS One 2014;9(4):e94026. PMID:24718563
35. Schenk AM, Fremouw WJ, Keelan CM. Characteristics of college cyberbullies. Comput Human Behav 2013;29(6):2320-7. doi:10.1016/j.chb.2013.05.013
36. Romero AJ, Wiggs CB, Valencia C, Bauman S. Latina teen suicide and bullying. Hisp J Behav Sci 2013;35(2):159-73. doi:10.1177/0739986312474237
37. Alavi N, Roberts N, Sutton C, Axas N, Repetti L. Bullying victimization (being bullied) among adolescents referred for urgent psychiatric consultation: prevalence and association with suicidality. Can J Psychiatry 2015;60(10):427-31. PMID:26720189
38. Roberts N, Axas N, Nesdole R, Repetti L. Pediatric Emergency Department Visits for Mental Health Crisis: Prevalence of Cyber-Bullying in Suicidal Youth. Child Adolesc Social Work J 2016;33(5):469-72. doi:10.1007/s10560-016-0442-8
39. Cénat JM, Blais M, Hébert M, Lavoie F, Guerrier M. Correlates of bullying in Quebec high school students: The vulnerability of sexual-minority youth. J Affect Disord 2015;183:315-21. PMID:26047959
40. Hébert M, Cénat JM, Blais M, Lavoie F, Guerrier M. Child sexual abuse, bullying, cyberbullying, and mental health problems among high schools students: a moderated mediated model. Depress Anxiety 2016;33(7):623-9. PMID:27037519
41. Hay C, Meldrum R, Mann K. Traditional bullying, cyber bullying, and deviance: A general strain theory approach. J Contemp Crim Justice 2010b;26(2):130-47. doi:10.1177/1043986209359557
42. Messias E, Kindrick K, Castro J. School bullying, cyberbullying, or both: Correlates of teen suicidality in the 2011 CDC youth risk behavior survey. Compr Psychiatry 2014;55(5):1063-8. PMID:24768228
43. Reed KP, Nugent W, Cooper RL. Testing a path model of relationships between gender, age, and bullying victimization and violent behavior, substance abuse, depression, suicidal ideation, and suicide attempts in adolescents. Child Youth Serv Rev 2015;55:128-37. doi:10.1016/j.childyouth.2015.05.016
44. Kindrick K, Castro J, Messias E. Sadness, suicide, and bullying in Arkansas: results from the Youth Risk Behavior Survey--2011. J Ark Med Soc 2013;110(5):90-1. PMID:24383197
45. Yen C-F, Chou W-J, Liu T-L, Ko C-H, Yang P, Hu H-F. Cyberbullying among male adolescents with attention-deficit/hyperactivity disorder: Prevalence, correlates, and association with poor mental health status. Res Dev Disabil 2014;35(12):3543-53. PMID:25241113
46. Arat G. Emerging protective and risk factors of mental health in Asian American students: findings from the 2013 Youth Risk Behavior Survey. Vulnerable Child Youth Stud 2015;10(3):192-205. doi:10.1080/17450128.2015.1045437

47. DeSmet A, Deforche B, Hublet A, Tanghe A, Stremersch E, De Bourdeaudhuij I. Traditional and cyberbullying victimization as correlates of psychosocial distress and barriers to a healthy lifestyle among severely obese adolescents – a matched case–control study on prevalence and results from a cross-sectional study. BMC Public Health 2014;14(1):224. PMID:24593118
48. Duong J, Bradshaw C. Associations between bullying and engaging in aggressive and suicidal behaviors among sexual minority youth: The moderating role of connectedness. J Sch Health 2014;84(10):636-45. PMID:25154527
49. Elgar FJ, Napoletano A, Saul G, Dirks MA, Craig W, Poteat VP, Holt M, Koenig BW. Cyberbullying victimization and mental health in adolescents and the moderating role of family dinners. JAMA Pediatr 2014;168(11):1015-22. PMID:25178884
50. Kodish T, Herres J, Shearer A, Atte T, Fein J, Diamond G. Bullying, depression, and suicide risk in a pediatric primary care sample. Crisis 2016. PMID:27040126
51. Litwiller BJ, Brausch AM. Cyber bullying and physical bullying in adolescent suicide: the role of violent behavior and substance use. J Youth Adolesc 2013;42(5):675-84. PMID:23381779
52. Mitchell KJ, Wells M, Priebe G, Ybarra ML. Exposure to websites that encourage self-harm and suicide: Prevalence rates and association with actual thoughts of self-harm and thoughts of suicide in the United States. J Adolesc 2014;37(8):1335-44. PMID:25313930
53. Roh B-R, Yoon Y, Kwon A, Oh S, Lee SI, Ha K, Shin YM, Song J, Park EJ, Yoo H. The structure of co-occurring bullying experiences and associations with suicidal behaviors in Korean adolescents. PLoS One 2015;10(11):e0143517. PMID:26619356
54. Sampasa-Kanyinga H, Roumeliotis P, Xu H. Associations between cyberbullying and school bullying victimization and suicidal ideation, plans and attempts among Canadian schoolchildren. PLoS One 2014;9(7):e102145. PMID:25076490
55. Sinclair KO, Bauman S, Poteat VP, Koenig B, Russell ST. Cyber and bias-based harassment: Associations with academic, substance use, and mental health problems. J Adolesc Health 2012;50(5):521-3. PMID:22525118
56. Turner MG, Exum ML, Brame R, Holt TJ. Bullying victimization and adolescent mental health: General and typological effects across sex. J Crim Justice 2013;41(1):53-9. doi:10.1016/j.jcrimjus.2012.12.005
57. Bonanno RA, Hymel S. Cyber bullying and internalizing difficulties: Above and beyond the impact of traditional forms of bullying. J Youth Adolesc 2013;42(5):685-97. PMID:23512485
